# Predictive value of plasma p-tau217 for Alzheimer’s dementia compared to other blood-based biomarkers

**DOI:** 10.1038/s44321-026-00483-9

**Published:** 2026-07-16

**Authors:** Kira Trares, Deniz Duman, Léon Beyer, Johannes C Michaelian, Hannah Stocker, Bernd Holleczek, Konrad Beyreuther, Ben Schöttker, Klaus Gerwert, Hermann Brenner

**Affiliations:** 1 Division of Clinical Epidemiology of Early Cancer Detection, German Cancer Research Center, Im Neuenheimer Feld 581, 69120 Heidelberg, Germany; 2 Center for Protein Diagnostics (ProDi), Ruhr-University Bochum, Bochum, Germany; 3 Healthy Brain Ageing Program, Brain and Mind Centre and Charles Perkins Centre, School of Psychology, Faculty of Science, University of Sydney, Sydney, New South Wales Australia; 4 Saarland Cancer Registry, Neugeländstraße 9, 66117 Saarbrücken, Germany; 5 Network Aging Research, Heidelberg University, Bergheimer Straße 20, 69115 Heidelberg, Germany; 6 Cancer Prevention Graduate School, German Cancer Research Center, Im Neuenheimer Feld 280, 69120 Heidelberg, Germany

**Keywords:** Biomarkers, Neuroscience

## Abstract

Phosphorylated-tau (p-tau217) is a promising blood-based biomarker for Alzheimer’s dementia (AD) in clinical settings. However, research from prospective cohort studies is sparse. We measured plasma p-tau217 levels in baseline blood samples of 779 participants in a population-based cohort of older adults followed over 17 years. Associations with AD were assessed and compared to those with previous measurements of p-tau181, neurofilament light chain (NfL), and glial fibrillary acidic protein (GFAP). Comparisons to the amyloid beta (Aβ) misfolding biomarker were performed in a subgroup analysis. P-tau217, NfL and GFAP showed strong associations with AD risk, especially within the first 9 years of follow-up, outperforming p-tau181. Over the later years of follow-up, the predictive accuracy of p-tau217 was significantly reduced. In contrast, the Aβ misfolding biomarker demonstrated superior performance especially as a preclinical indicator of the risk of AD many years before diagnosis. The combination of p-tau217 with NfL, GFAP, basic demographic and genetic information, as well as the misfolding biomarker yielded an AUC 0.86 for AD diagnoses over the entire 17-year follow-up period.

## Synopsis

P-tau217 is a promising blood-based biomarker for Alzheimer’s dementia (AD) in the clinical stage. In this prospective cohort study, it showed better performance in the prodromal than in the preclinical stage up to 17 years before diagnosis.

- In a subgroup analysis, the Aß misfolding biomarker showed higher discriminative ability in the preclinical stage compared to p-tau217.
- A prediction model including p-tau217, NfL, GFAP, Aß misfolding as well as basic demographic and genetic information reached an AUC of 0.86 for AD over the entire follow-up period.

## Abstract (web summary)

P-tau217 is a promising blood-based biomarker for Alzheimer’s dementia (AD) in the clinical stage. In this prospective cohort study, it showed better performance in the prodromal than in the preclinical stage up to 17 years before diagnosis.

## Graphical abstract

**Figure:**
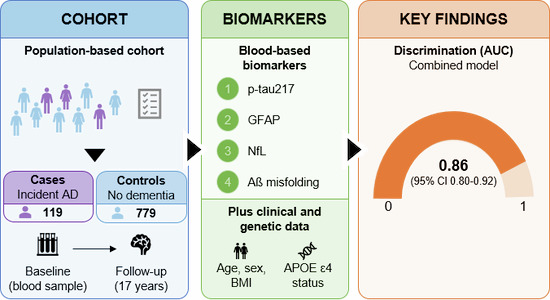

**Figure:**
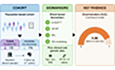

The paper explained **Problems**

Currently, therapeutic antibodies that slow the progression of Alzheimer’s dementia (AD) have been approved by the FDA and EMA. However, the response to these therapies is often limited, and serious side effects can occur. Additionally, only a specific subgroup of patients qualifies for treatment. This underscores the importance of identifying individuals at risk of AD at a preclinical, symptom-free stage and highlights the need for blood-based biomarkers to identify these individuals.

**Results**

In this study, we show that the biomarker p-tau217 is strongly associated only with the clinical stage of AD, similar to established biomarkers such as the abeta 42/40 ratio, but shows only low performance in the preclinical stage up to 17 years before clinical diagnosis. In contrast, the Aß misfolding biomarker showed stronger performance at preclinical stages up to 17 years before diagnosis. The combination of this misfolding biomarker with basic demographic and genetic information, as well as the other blood-based biomarkers, improved disease prediction accuracy.

**Impact**

The information included in this model can be easily obtained and suggests potential as a cost-effective and scalable tool for preclinical screening of the elderly population.

## Introduction

The global incidence of dementia continues to rise and is projected to reach 78 million cases by 2030 (WHO, 2021). While disease-modifying medications are now accessible for mild cognitive impairment (MCI) and early-stage Alzheimer’s dementia (AD), early identification of individuals at risk of dementia and AD is crucial, particularly before clinical AD stages manifest (Teunissen et al, 2022). However, current diagnostic procedures, such as cerebrospinal fluid (CSF) tests like the amyloid-ß (Aβ) 42/40 ratio and imaging techniques like Aβ positron emission tomography (PET), are costly, invasive and primarily indicate only a large amyloid burden in the clinical stage (Thijssen et al, 2020). In recent years, advancements in blood biomarkers for AD have emerged, showing promise for clinical application (Teunissen et al, 2022; Teunissen et al, 2020). These markers have the potential to facilitate preclinical diagnoses, monitor treatment efficacy, and track disease progression (Ossenkoppele et al, 2022; Ashton et al, 2024; Pereira et al, 2021).

Over the past few years, p-tau217 has been proposed as a promising blood-based biomarker for clinical AD, demonstrating significant associations with progressive cognitive decline and AD (Mattsson-Carlgren et al, 2023; Palmqvist et al, 2023; Janelidze et al, 2022; Brickman et al, 2021; Groot et al, 2022). Compared to other p-tau proteins like p-tau181 and p-tau231, p-tau217 has been found to exhibit stronger associations with AD (Janelidze et al, 2022; Bayoumy et al, 2021; Ashton et al, 2023), reaching discriminative accuracies of up to 92% (Ashton et al, 2023). Notably, findings are consistent with CSF measurements (Ashton et al, 2024; Ashton et al, 2023; Janelidze et al, 2022; Therriault et al, 2023) and reveal good discrimination between AD and other neurological disorders (Ashton et al, 2023; Groot et al, 2022). However, although findings have been replicated across numerous studies in clinical settings, including different ethnicities (Xiao et al, 2023; Brickman et al, 2021), evidence from cohort studies with blood p-tau measurements in the preclinical stage taken years prior to clinical AD diagnoses is still sparse. Nevertheless, this is urgently needed to assess the biomarker performance for AD risk assessment and early detection (Teunissen et al, 2022; Ossenkoppele et al, 2022; Xiao et al, 2023) as well as for prevention and early therapy, when the brain is less affected by amyloid burden. In addition to phosphorylated tau biomarkers, neurofilament light chain (NfL), glial fibrillary acidic protein (GFAP), and Aβ misfolding are well-established biomarkers in the field. NfL is a general marker of neurodegeneration associated with various neurodegenerative conditions, including AD (Teunissen et al, 2022; Garcia-Escobar et al, 2024). On the other hand, GFAP is a marker of astrocyte activation, indicating near-clinical AD pathology (Stocker et al, 2023a; Verberk et al, 2021; Beyer et al, 2023). Lastly, the early misfolding of Aβ in blood is an established structure-based biomarker for AD risk in the preclinical stage up to 17 years before diagnosis and may help predict future cognitive decline (Nabers et al, 2016; Nabers et al, 2019; Nabers et al, 2018). In combination, GFAP and misfolded Aβ achieved an AUC of 0.828 (95% CI: 0.764–0.891) for discrimination of AD up to 17 years before diagnosis (Beyer et al, 2023).

This study aims to examine the association of p-tau217 with AD and other forms of dementia (i.e., all-cause dementia, vascular dementia, and mixed dementia) and to assess their predictive ability in a large, German prospective cohort study followed over 17 years. Additionally, this research aims to compare the predictive performance of p-tau217 with other blood-based biomarkers, including the structure-based Aß misfolding biomarker recently discussed in the literature.

## Results

**Figure 1:**
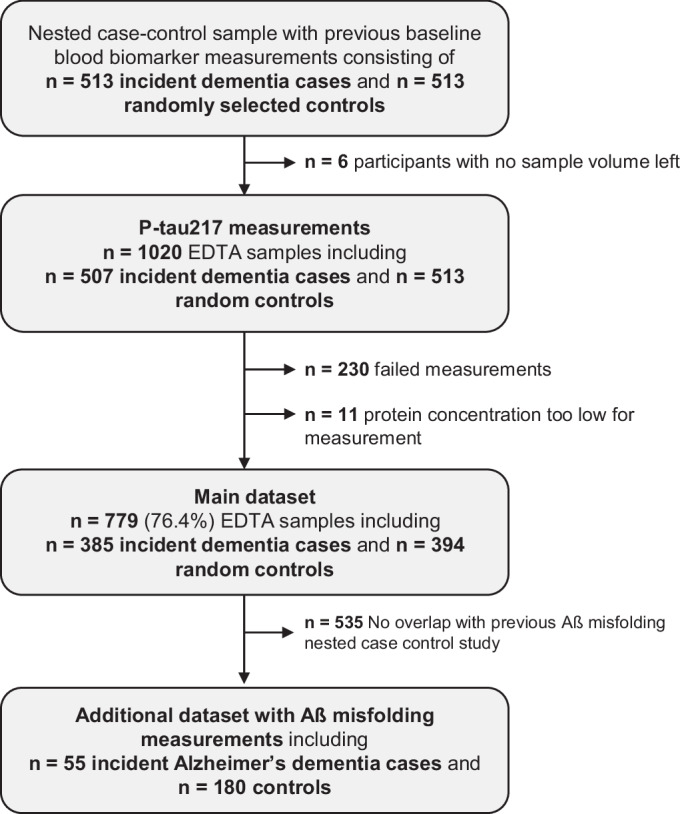
Flowchart of the study population for this research project. GP general practitioner, Aß amyloid beta.

Baseline characteristics of study participants are displayed separately for incident all-cause dementia and AD cases as well as controls in Table 1. Corresponding data for VD and MD cases, along with controls, are shown in Appendix Table S1. Among the included study participants, 385 received a dementia diagnosis during a median follow-up time of 13.6 years (interquartile range (IQR): 9.7–16.7 years) (Fig. 1). Of those, GPs reported 122 to be specifically diagnosed with AD, 146 with VD, and 66 with MD. At baseline, the mean age of study participants was ~6 years higher in participants with incident all-cause dementia (mean [SD]: 67.0 [5.2] years) compared to controls (61.4 [6.5] years). In addition, most participants had an education of up to 9 years (>80%), reported a low level of physical activity, were never smokers and stated a low or moderate consumption of alcohol. The proportion of *APOE* ε4 carriers was markedly higher among participants diagnosed with dementia, especially AD (47.8%), compared to participants with no dementia diagnosis (26.8%).

**Table 1:** Baseline characteristics of included study participants (*n* = 779).

Baseline characteristics	Controls (*n* = 394)	Cases	
		All-cause dementia (*n* = 385)	Alzheimer’s dementia (*n* = 122)
Age (years), mean (SD)	61.4 (6.5)	67.0 (5.2)	66.4 (5.1)
Sex, *n* (%)			
Female	213 (54.1)	199 (51.7)	70 (57.4)
Male	181 (45.9)	186 (48.3)	52 (42.6)
Education			
≤9	309 (80.3)	299 (81)	101 (84.9)
10–11	39 (10.1)	37 (10)	9 (7.6)
≥12	37 (9.6)	33 (8.9)	9 (7.6)
BMI (kg/m^2^)			
<25	97 (24.7)	119 (31)	42 (34.7)
25–30	183 (46.7)	171 (44.5)	53 (43.8)
≥30	112 (28.6)	94 (24.5)	26 (21.5)
Physical activity^a^			
Inactive	71 (18)	115 (30)	40 (32.8)
Low	176 (44.7)	166 (43.2)	48 (39.3)
Medium or high	147 (37.3)	103 (26.8)	34 (27.9)
Diabetes			
No	338 (86.2)	299 (78.5)	98 (80.3)
Yes	54 (13.8)	82 (21.5)	24 (19.7)
CVD^b^			
No	312 (79.2)	269 (69.9)	94 (77.1)
Yes	82 (20.8)	116 (30.1)	28 (23.0)
Smoking status			
Never smoker	199 (51.2)	211 (56.7)	70 (59.3)
Former smoker	128 (32.9)	119 (32.0)	35 (29.7)
Current smoker	62 (15.9)	42 (11.3)	13 (11)
Alcohol consumption^c^			
None	118 (31.7)	113 (34.9)	37 (35.9)
Low or moderate	221 (59.4)	193 (59.6)	59 (57.3)
High	22 (8.9)	18 (5.6)	7 (6.8)
Life-time history of depression			
No	338 (85.8)	324 (84.2)	107 (87.7)
Yes	56 (14.2)	61 (15.8)	15 (12.3)
Kidney function			
eGFRcr-cys, mean (SD), mL/min/1.73m^2^	83.8 (16.1)	79.8 (16.3)	79.5 (16.9)
eGFRcr-cys, mL/min/1.73^2^			
≥90	139 (35.3)	106 (27.5)	33 (27.1)
60–89	222 (56.4)	236 (61.3)	73 (59.8)
<60	33 (8.4)	43 (11.2)	16 (13.1)
*APOE* ε4 carrier status			
Non-carrier	282 (73.3)	216 (59.7)	60 (52.2)
Carrier	103 (26.8)	146 (40.3)	55 (47.8)

*SD* standard deviation, *BMI* body mass index, *CVD* cardiovascular disease, *APOE* apolipoprotein E.

^a“^Inactive” was defined by <1 h of vigorous or <1 h of light physical activity per week. “Medium or high” was defined by ≥2 h of vigorous and ≥2 h of light physical activity per week. All other amounts of physical activity were grouped into the category “Low”.

^b^CVD was defined as diagnosed coronary heart disease or self-reported history of myocardial infarction, stroke, pulmonary embolism, or coronary artery revascularization.

^c^Low or moderate alcohol consumption was defined by 0 to 19.99 g ethanol/day (g/d) for women and 0 to 39.99 g/d for men; high alcohol consumption was defined by ≥20 to 39.99 g/d for women and ≥40 g/d for men.

### All-cause dementia and Alzheimer’s dementia

**Figure 2:**
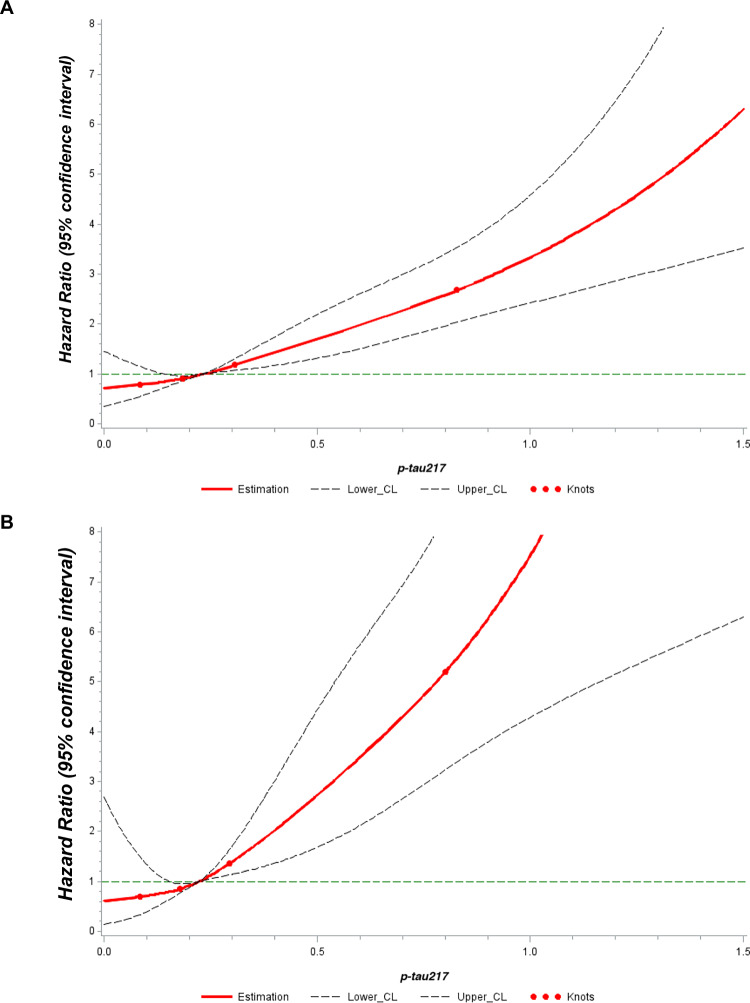
Dose–response relationship between baseline p-tau217 and incident dementia. Dose-response relationship between p-tau217 biomarker levels at baseline and incident (**A**) all-cause dementia and (**B**) AD diagnoses within 17 years in a spline regression model. Knots were placed at the 5th, 35th, 65th, and 90th percentiles of p-tau217 levels. Case numbers: all *n* = 775; (**A**) all-cause dementia cases: *n* = 381; (**B**) AD cases: *n* = 121. Source data are available online for this figure.

P-tau217 levels were significantly higher among all-cause dementia and AD cases compared to controls (*p* < 0.0001, Appendix Figs. S1A and S1B). In addition, the dose-response curves showed a steadily increasing all-cause dementia risk with increasing p-tau217 levels, especially above the 35th percentile (Fig. 2A). Nevertheless, the increase was distinctly stronger for AD (Fig. 2B).

Cox regression models revealed statistically significant associations between log-transformed p-tau217 levels and the risk of all-cause dementia during follow-up. However, this association was observed within the first 9 years only and weaker (HR (95%CI) per 1 SD increase in the multivariate model: 1.58 (1.16–2.16)) than the association observed for GFAP (1.95 (1.25–3.04) (Table 2). None of the biomarkers was associated with all-cause dementia in the later years of follow-up (9–17 years) in the multivariate analysis.

**Table 2:** Cox Regression results. HRs for incident all-cause dementia diagnosis stratified by time of diagnosis.

	Model 1^a^				Model 2^b^				Model 3^c^			
	n_total_	n_cases_	HR (95% CI)	*P* value^d^	n_total_	n_cases_	HR (95% CI)	*P* value^d^	n_total_	n_cases_	HR (95% CI)	*P* value^d^
**0–9 years**												
p-tau217	511	121	**1.98 (1.57–2.50)**	**<0.0001**	477	104	**1.43 (1.05–1.93)**	**0.0225**	432	87	**1.58 (1.16–2.16)**	**0.0041**
p-tau181	511	121	**1.42 (1.14–1.77)**	**0.0017**	477	104	1.01 (0.78–1.32)	0.9391	432	87	1.04 (0.72–1.49)	0.8366
NfL	508	121	**1.99 (1.68–2.36)**	**<0.0001**	474	104	**1.34 (1.07–1.68)**	**0.0121**	429	87	**1.44 (1.08–1.91)**	**0.0119**
GFAP	508	121	**2.51 (1.96–3.22)**	**<0.0001**	474	104	**1.66 (1.15–2.40)**	**0.0073**	429	87	**1.95 (1.25–3.04)**	**0.0031**
**9–17 years**												
p-tau217	640	250	**1.34 (1.14–1.57)**	**0.0004**	601	228	1.13 (0.94–1.37)	0.2034	536	191	1.10 (0.89–1.36)	0.3910
p-tau181	640	250	1.04 (0.87–1.23)	0.6960	601	228	0.93 (0.79–1.08)	0.3308	536	191	0.87 (0.74–1.02)	0.0795
NfL	635	248	**1.48 (1.30–1.69)**	**<0.0001**	596	226	1.01 (0.85–1.21)	0.8779	531	189	0.94 (0.77–1.15)	0.5726
GFAP	635	248	**1.74 (1.45–2.10)**	**<0.0001**	596	226	1.22 (0.94–1.58)	0.1350	531	189	1.28 (0.94–1.75)	0.1195
**0–17 years**												
p-tau217	761	371	**1.47 (1.29–1.68)**	**<0.0001**	705	332	**1.22 (1.05–1.43)**	**0.0118**	623	278	**1.26 (1.05–1.47)**	**0.0129**
p-tau181	761	371	**1.14 (1.94–1.30)**	**0.0428**	705	332	0.98 (0.87–1.11)	0.7521	623	278	0.94 (0.82–1.08)	0.4004
NfL	756	369	**1.62 (1.45–1.80)**	**<0.0001**	700	330	1.13 (0.99–1.30)	0.0748	618	276	1.10 (0.93–1.29)	0.2611
GFAP	756	369	**1.80 (1.55–2.08)**	**<0.0001**	700	330	**1.28 (1.05–1.56)**	**0.0166**	618	276	**1.37 (1.09–1.74)**	**0.0081**

Numbers printed in bold are statistically significant (*p* < 0.05). All biomarker levels are per 1 SD increase in natural log-transformed biomarker level.

*HR* hazard ratio, *CI* confidence interval.

^a^Model 1: crude model.

^b^Model 2: adjusted for age, sex, education, and *APOE* ε4 carrier status.

^c^Model 3: multivariable model adjusted for age, sex, education, BMI, physical activity, diabetes, CVD risk, life-time history of depression, kidney function, alcohol consumption, smoking, and *APOE* ε4 carrier status.

^d^Results of Wald test.

Similar patterns, but generally stronger associations were seen between the biomarkers and AD compared to all-cause dementia (Table 3). However, associations with AD diagnoses in later years were weaker than for diagnoses in earlier years for all biomarkers and remained statistically significant for GFAP and p-tau217 only (HR (95% CI) in the multivariate model: 1.80 (1.22–2.65) and 1.63 (1.20–2.20), respectively). These two biomarkers also showed the best discriminative ability over the entire follow-up (HR (95% CI) in multivariate model: 1.75 (1.20–2.55) and 1.82 (1.39–2.39), respectively).

**Table 3:** Cox regression results. HRs for incident Alzheimer’s dementia diagnosis stratified by time of diagnosis.

	Model 1^a^				Model 2^b^				Model 3^c^			
	*n*_total_	*n*_cases_	HR (95% CI)	*P* value^d^	*n*_total_	*n*_cases_	HR (95% CI)	*P* value^d^	*n*_total_	*n*_cases_	HR (95% CI)	*P* value^d^
**0–9 years**												
p-tau217	432	42	**3.11 (2.14–4.54)**	**<0.0001**	411	38	**2.12 (1.33–3.37)**	**0.0015**	377	32	**2.52 (1.40–4.55)**	**0.0022**
p-tau181	432	42	**2.00 (1.48–2.69)**	**<0.0001**	411	38	1.41 (0.92–2.18)	0.1176	377	32	1.54 (0.95–2.50)	0.0779
NfL	429	42	**2.43 (1.87–3.17)**	**<0.0001**	408	38	**1.75 (1.21–2.52)**	**0.0028**	374	32	**2.04 (1.16–3.58)**	**0.0129**
GFAP	429	42	**3.02 (1.74–5.23)**	**<0.0001**	408	38	1.94 (0.87–4.34)	0.1060	374	32	2.06 (0.77–5.50)	0.1505
**9–17 years**												
p-tau217	467	77	**1.85 (1.45–2.36)**	**<0.0001**	444	71	**1.53 (1.16–2.02)**	**0.0029**	404	59	**1.63 (1.20–2.20)**	**0.0015**
p-tau181	467	77	1.30 (0.95-1.79)	0.1035	444	71	1.14 (0.81-1.61)	0.4632	404	59	1.08 (0.72–1.63)	0.6987
NfL	464	77	**1.66 (1.33–2.06)**	**<0.0001**	441	71	1.21 (0.89–1-64)	0.2206	401	59	1.14 (0.78–1.66)	0.4988
GFAP	464	77	**2.19 (1.65–2.90)**	**<0.0001**	441	71	**1.67 (1.17–2.39)**	**0.0045**	401	59	**1.80 (1.22–2.65)**	**0.0030**
**0–17 years**												
p-tau217	509	119	**2.15 (1.74–2.66)**	**<0.0001**	482	109	**1.71 (1.34–2.19)**	**<0.0001**	436	91	**1.82 (1.39–2.39)**	**<0.0001**
p-tau181	509	119	**1.49 (1.20–1.87)**	**0.0003**	482	109	1.20 (0.93–1.55)	0.1552	436	91	1.19 (0.89–1.58)	0.2470
NfL	506	119	**1.92 (1.61–2.32)**	**<0.0001**	479	109	**1.36 (1.06–1.75)**	**0.0166**	433	91	**1.39 (1.01–1.89)**	**0.0450**
GFAP	506	119	**2.29 (1.77–2.95)**	**<0.0001**	479	109	**1.62 (1.16–2.27)**	**0.0046**	433	91	**1.75 (1.20–2.55)**	**0.0033**

Numbers printed in bold are statistically significant (*p* < 0.05). All biomarker levels are per 1 SD increase in natural log-transformed biomarker level.

*HR* hazard ratio, *CI* confidence interval.

^a^Model 1: crude model.

^b^Model 2: adjusted for age, sex, education, and *APOE* ε4 carrier status.

^c^Model 3: multivariable model adjusted for age, sex, education, BMI, physical activity, diabetes, CVD risk, life-time history of depression, kidney function, alcohol consumption, smoking, and *APOE* ε4 carrier status.

^d^Results of Wald test.

**Figure 3:**
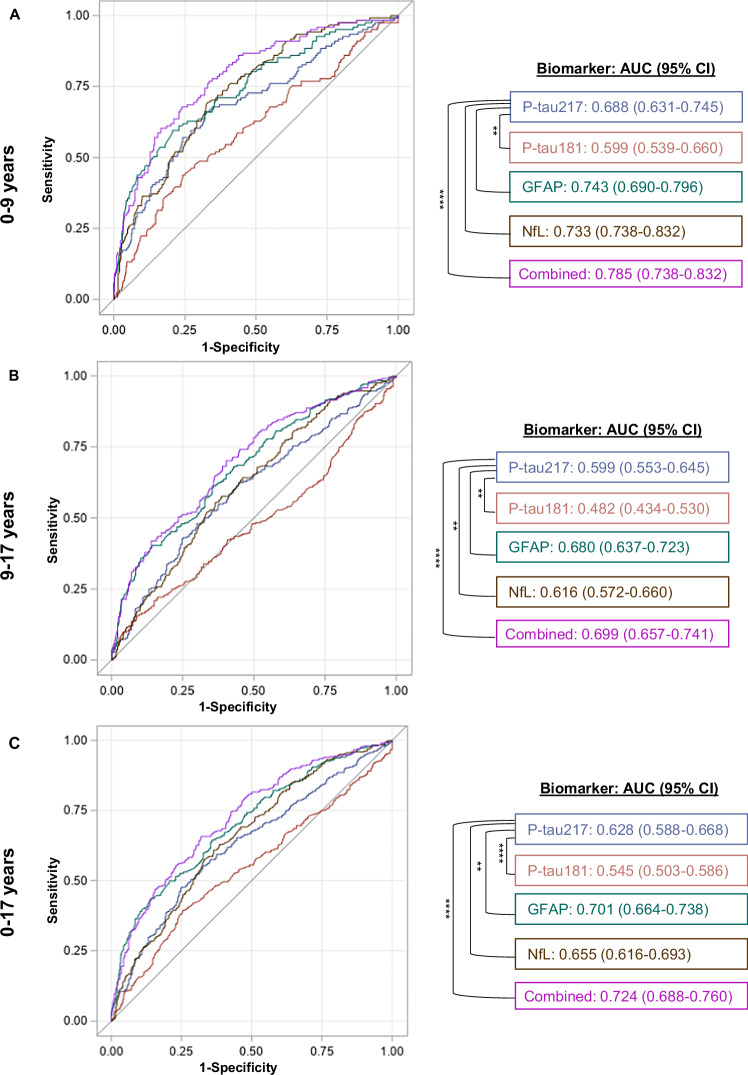
ROC curves of baseline biomarkers for incident all-cause dementia. ROC curves and contrast using the DeLong test for incident all-cause dementia within (**A**) 0–9 years, (**B**) 9–17 years, and (**C**) 0–17 years of follow-up based on baseline biomarker levels. Biomarker levels are natural log-transformed levels from baseline. *P < 0.05, **P < 0.01, ***P < 0.001, ****P < 0.0001 (DeLong Test). Case numbers: controls: *n* = 387; all-cause dementia_0–9 years_ *n* = 121; all-cause dementia_9–17 years_ *n* = 248; all-cause dementia_0–17 years_ *n* = 369. Source data are available online for this figure.

The discriminative accuracy of log-transformed p-tau217 levels for incident all-cause dementia was 0.688, 0.599, and 0.628 for the first 9 years, between 9 and 17 years, and the whole follow-up period, respectively (Fig. 3A–C). P-tau217 performed significantly better compared to p-tau181 (*p* < 0.0001, DeLong Test), but worse than GFAP and NfL, reaching AUCs of up to 0.743 and 0.733 in the first 9 years of follow-up, respectively. However, combining p-tau217 with the other blood-based biomarkers resulted in the highest AUCs for the outcome of all-cause dementia.

**Figure 4:**
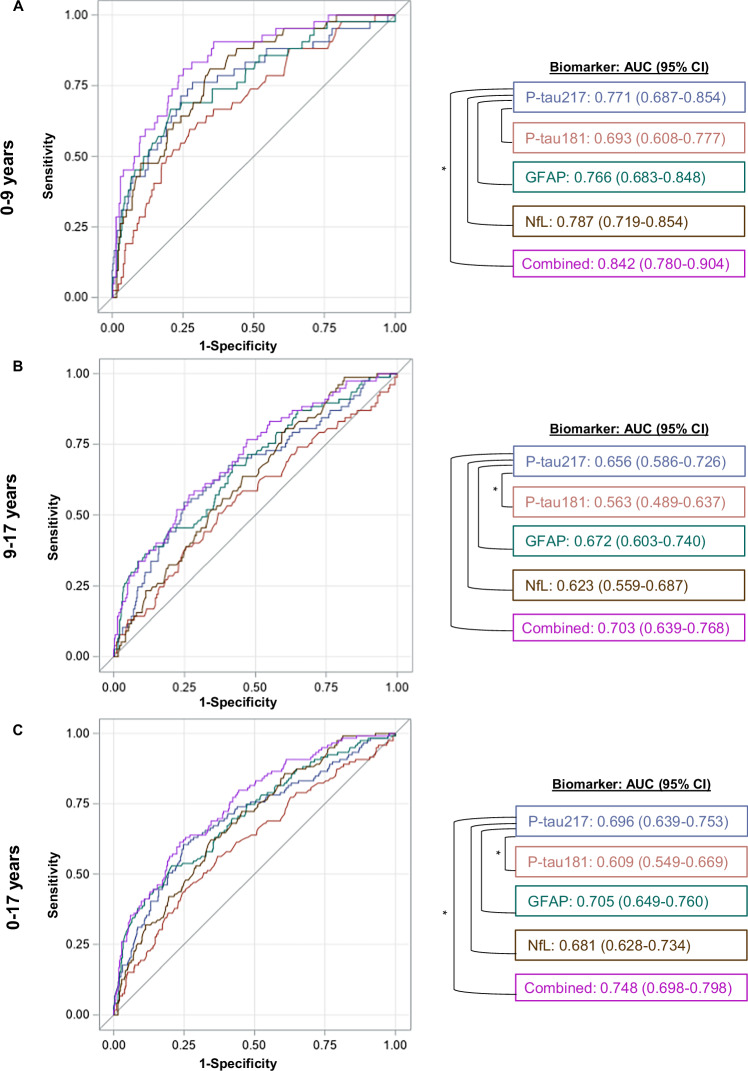
ROC curves of baseline biomarkers for incident Alzheimer’s dementia. ROC curves and contrast using the DeLong test for incident Alzheimer’s dementia within (**A**) 0–9 years, (**B**) 9–17 years, and (**C**) 0–17 years of follow-up based on baseline biomarker levels. Biomarker levels are natural log-transformed levels from baseline. *P < 0.05, **P < 0.01, ***P < 0.001, ****P < 0.0001 (DeLong Test). Case numbers: controls: *n* = 387; AD_0–9 years_ *n* = 42; AD_9–17 years_ *n* = 77; AD_0–17 years_ *n* = 119. Source data are available online for this figure.

For AD, all biomarkers showed better performance than for all-cause dementia (Fig. 4A–C). In the prodromal stage (0–9 years, Fig. 4A), AUCs were higher than those for the preclinical stage (9–17 years, Fig. 4B); especially, the p-tau217 marker performance decreased to an AUC of 0.656 (Fig. 4B).

P-tau217 performed once more significantly better compared to p-tau181, and achieved comparable AUCs to GFAP and NFL. Combining all four blood-based biomarkers increased the discriminative performance for AD (0–9 years: AUC = 0.842; 9–17 years: AUC = 0.703; 0–17 years: AUC = 0.748). In a sensitivity analysis excluding p-tau181 from the combined model, the AUC remained unchanged, indicating that p-tau181 did not contribute meaningfully to the model’s discriminative ability (Appendix Fig. S5).

**Figure 5:**
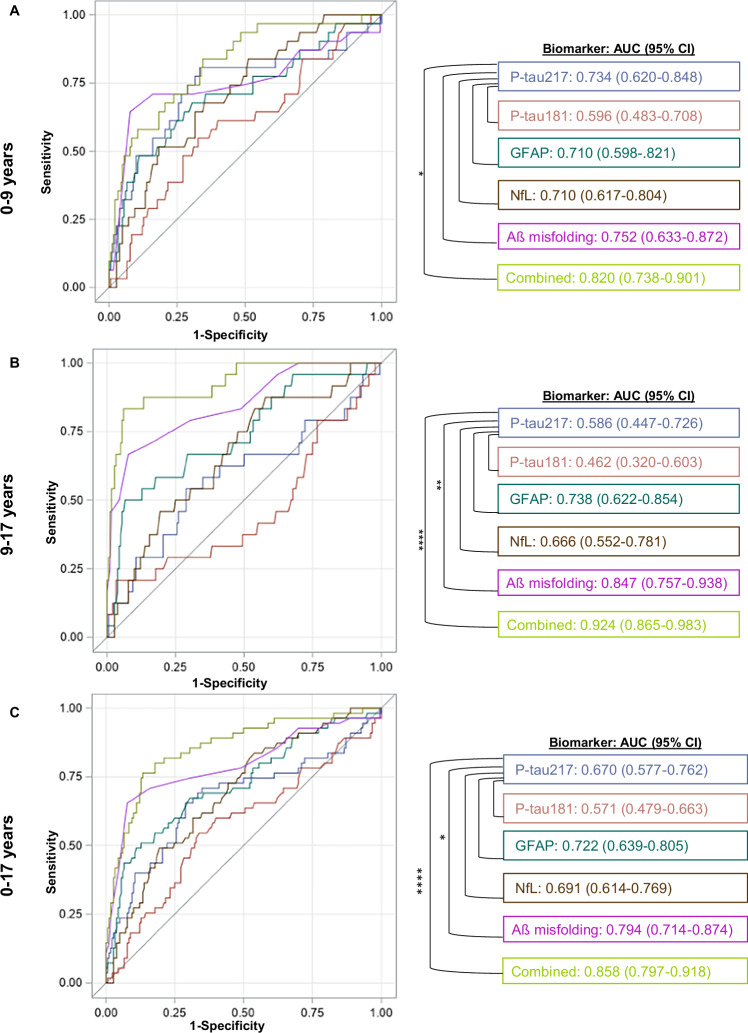
ROC curves of baseline biomarkers for incident Alzheimer’s disease in the Aβ misfolding subcohort. ROC curves and contrast using the DeLong test for incident Alzheimer’s dementia within (**A**) 0–9 years, (**B**) 9–17 years, and (**C**) 0–17 years of follow-up based on baseline biomarker levels. Biomarker levels are natural log-transformed levels from baseline. *P < 0.05, **P < 0.01, ***P < 0.001, ****P < 0.0001 (DeLong Test). Case numbers: controls: *n* = 180; AD_0–9 years_ *n* = 31; AD_9–17 years_ *n* = 24; AD_0–17 years_ *n* = 55. Source data are available online for this figure.

To directly compare p-tau217 and the Aβ misfolding biomarker, we conducted a subgroup analysis including only participants with available measurements for both biomarkers (*n* = 55 AD cases, *n* = 180 controls) (Fig. 5A–C). In this subgroup, the Aβ misfolding biomarker showed significantly higher discriminative ability than p-tau217, especially in the preclinical phase (9–17 years: AUC = 0.847 vs. 0.586, Fig. 5B) and across the entire follow-up period (AUC = 0.794 vs. 0.670, Fig. 5C), while both biomarkers performed comparably within the prodromal phase (0–9 years: AUC = 0.752 vs. 0.734, Fig. 5A).

Inclusion of the Aβ misfolding biomarker to the p-tau217, p-tau181, GFAP, and NfL biomarker panel further improved predictive performance in this subgroup, particularly during the preclinical phase (Fig. 5).

**Figure 6:**
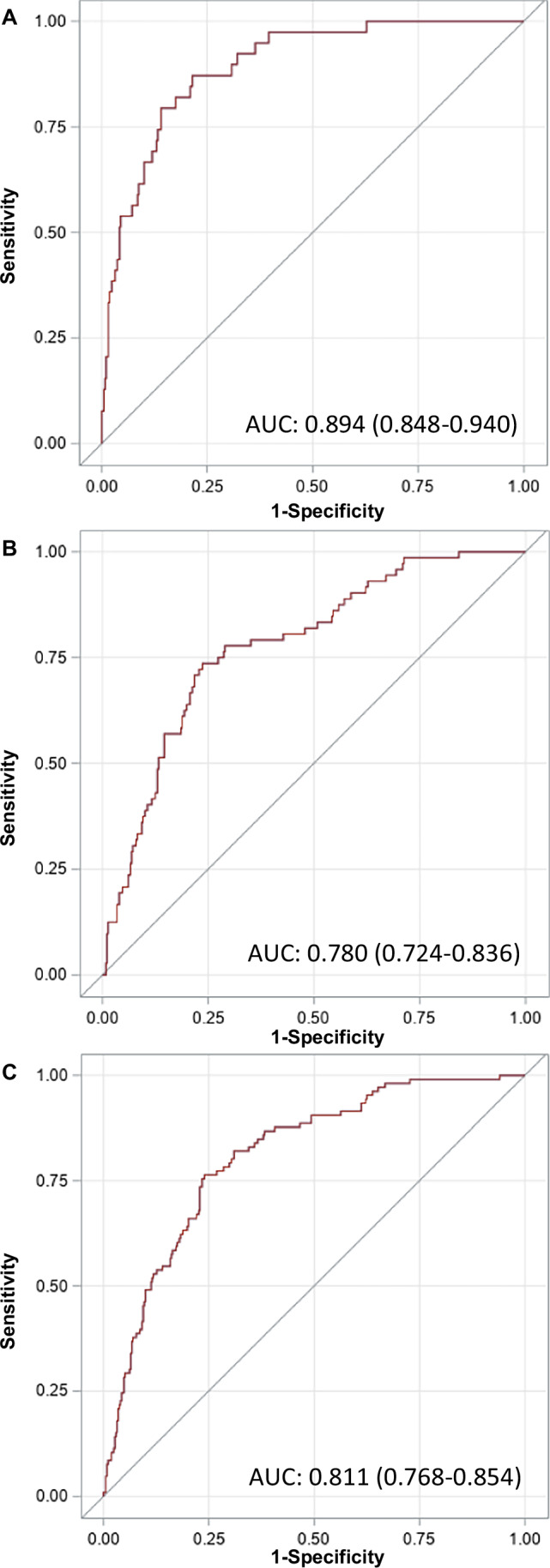
ROC curves of baseline biomarkers, demographic and genetic variables for incident Alzheimer’s disease. ROC curves for incident Alzheimer’s dementia within (**A**) 0–9 years, (**B**) 9–17 years, and (**C**) 0–17 years of follow-up based on baseline p-tau217, NfL, and GFAP levels, as well as age, sex, BMI, and APOE ε4 carrier status. Biomarker levels are natural log-transformed levels from baseline. Case numbers: controls: *n* = 376; AD_0–9 years_ *n* = 39; AD_9–17 years_ *n* = 72; AD_0–17 years_ *n *= 111. Source data are available online for this figure.

Adding demographic and genetic covariates (age, sex, BMI, and APOE ε4 carrier status) to the p-tau217, GFAP, and NfL biomarker panel increased the model performance during the prodromal stage (AUC = 0.894, Fig. 6A), but only modestly affected discriminative ability in the preclinical stage (AUC = 0.780, Fig. 6B) and across the full follow-up period (AUC = 0.810, Fig. 6C). The incremental performance of p-tau217 alone in this model was additionally depicted by delta AUC measures in Appendix Table S2.

**Figure 7:**
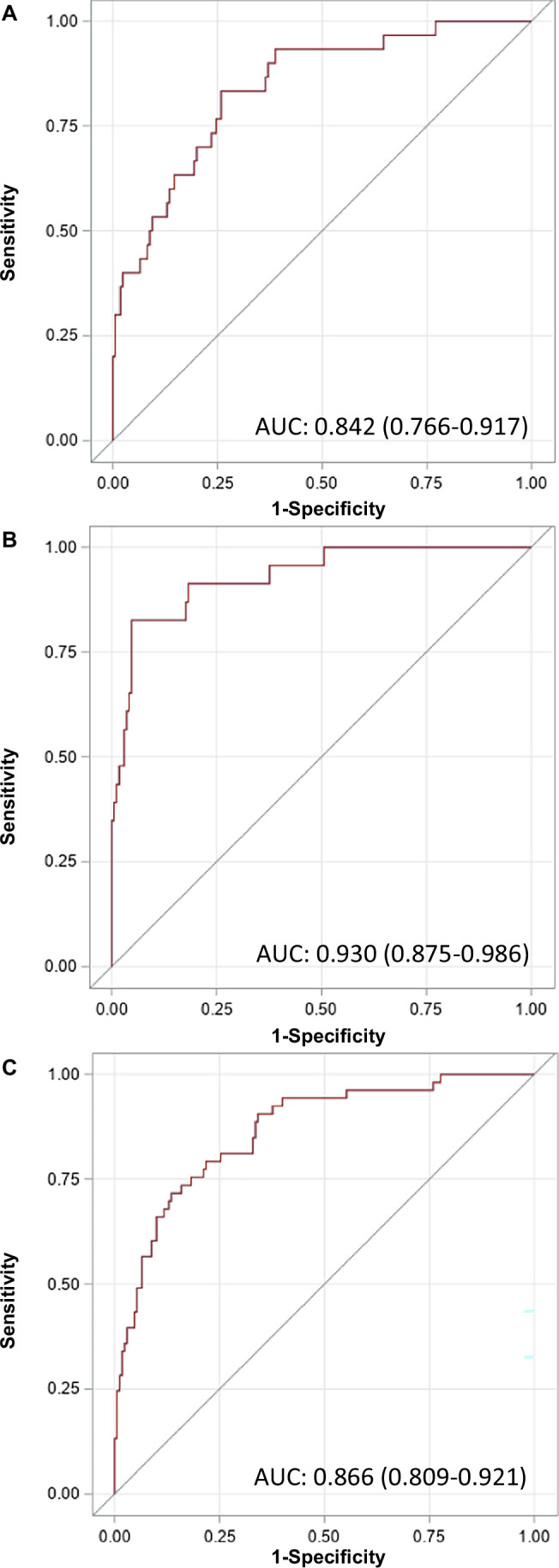
ROC curves of baseline biomarkers, Aβ misfolding, demographic and genetic variables for incident Alzheimer’s dementia. ROC curves for incident Alzheimer’s dementia within (**A**) 0–9 years, (**B**) 9–17 years and (**C**) 0–17 years of follow-up based on baseline p-tau217, NfL, GFAP, and Aß misfolding levels, as well as age, sex, BMI, and APOE ε4 carrier status. Biomarker levels are natural log-transformed levels from baseline. Case numbers: controls: *n* = 180; AD_0–9 years_ *n* = 30; AD_9–17 years _*n* = 23; AD_0–17 years _*n* = 53. Source data are available online for this figure.

Aβ misfolding in the extended model with demographic and genetic variables further improved model performance in this exploratory setting as compared to p-tau217, GFAP, and NfL, especially in the prediction of late cases (9–17 years) from 0.780 to 0.930 (Fig. 7A–C).

### Vascular and mixed dementia

Dose-response analyses showed an increasing risk of developing VD and MD with rising p-tau217 levels beyond the 35th percentile, a pattern similar to that observed for all-cause dementia and AD (Appendix Fig. S2). However, in regression models using log-transformed biomarker values per 1 SD increase, p-tau217 was not statistically significantly associated with VD, and its association with MD lost statistical significance after adjustment for confounders (Appendix Tables S3 and 4).

In contrast, GFAP was significantly associated with VD incidence during the first 9 years of follow-up (HR of 2.71 (95% CI: 1.44–5.09) - multivariate model) and with MD incidence, during the preclinical phase (9–17 years) as well as across the full follow-up duration. NfL and p-tau181 showed significant associations with MD during the first 9 years, and NfL as well over the entire follow-up period. Of note, the low number of cases for this outcome and time interval resulted in wide confidence intervals. Consistent with these results, GFAP showed the highest AUCs for VD, especially in the first 9 years of follow-up (AUC [95% CI]: 0.739 [0.649–0.829]) (Appendix Fig. S3) and NfL demonstrated the strongest discriminative performance for MD in the prodromal phase, with an AUC of 0.832 (95% CI: 0.751–0.913), significantly outperforming p-tau217 (*p* < 0.05; Appendix Fig. S4). The combination of all four blood-based biomarkers yielded only a modest improvement in the predictive accuracy for VD as well as for MD. Of note, GFAP displayed stable AUCs across all time periods for both VD and MD outcomes.

## Discussion

In this prospective cohort study, we examined the association of plasma p-tau217 with incident all-cause dementia, AD, VD, and MD, and evaluated its discriminative ability in comparison to other blood-based biomarkers. P-tau217, NfL and GFAP showed strong associations with the outcome of AD, especially in the prodromal stage, within the first 9 years of follow-up, outperforming p-tau181. Furthermore, p-tau217 demonstrated discriminative performance for AD during this stage, which improved further when combined with additional biomarkers (NfL and GFAP), as well as demographic and genetic factors (age, sex, BMI, and *APOE* ε4 status), finally achieving an AUC of 0.894. However, the preclinical phase and the entire time span, the discriminative accuracy of p-tau217 was significantly reduced. In contrast, the Aβ misfolding biomarker demonstrated superior performance in a subgroup analysis, especially as a preclinical indicator of the risk of AD.

### Previous studies

Only a few prospective cohort studies, including a comprehensive set of p-tau217, p-tau181, NfL, and GFAP measurements, enabling a head-to-head comparison of p-tau217 with other blood-based biomarkers, have been reported so far.

In the study by Grande et al, 2148 adults aged 60 and older were followed for up to 16 years as part of the Swedish National Study on Aging and Care in Kungsholmen (SNAC-K) (Grande et al, 2025). Higher quartiles of p-tau217, p-tau181, NfL, and GFAP were reported to be associated with increased risk of all-cause dementia and AD. For the outcome of all-cause dementia, p-tau217 showed comparable risk estimates to NfL and GFAP, outperforming p-tau181. For AD, NfL and GFAP yielded higher HRs than p-tau217, though with wider confidence intervals. In terms of 10-year discriminative ability, p-tau217 showed a discriminative ability of 0.810 for all-cause dementia and 0.768 for AD, the latter representing the highest discriminative ability among the biomarkers for this outcome. Combining p-tau217 with NfL and GFAP further improved the discrimination of AD to 0.813. While the findings of Grande et al align with ours in several respects, it is noteworthy that they consistently showed stronger results for all-cause dementia, despite 58% of dementia cases being AD, whereas p-tau217 performed distinctly better for the outcome of AD in our study. Additionally, although Grande et al adjusted for comorbidities, key risk and lifestyle factors such as BMI, physical activity, or smoking were not included in the multivariable Cox regression models.

Furthermore, Binette and colleagues performed measurements of blood-based biomarkers (p-tau217, p-tau181, NfL, GFAP, and Aβ42/Aβ40 ratio) in the Swedish BioFINDER-1 (*n* = 748) and the BioFINDER-2 (*n* = 421) cohorts (Pichet Binette et al, 2023). The authors evaluated several covariates for an association with blood-based biomarkers. In both cohorts, creatinine levels and BMI were consistently associated with GFAP and NfL, while elevated creatinine levels were associated with higher p-tau217 and p-tau181 levels only. Disease prediction models in the BioFINDER-1 cohort for 4 years of follow-up yielded AUCs for all-cause dementia of 0.73, 0.70, 0.71, and 0.70 for p-tau217, p-tau181, NfL, and GFAP, respectively. For AD, the corresponding AUCs were 0.82, 0.82, 0.67, and 0.74, respectively. A combination of p-tau217 with creatinine and BMI led to an improvement of 0.01 in the AUC for AD. Likewise, in our study, the inclusion of BMI in the prediction model yielded to an increase in AUC of 0.02.

A study conducted by Mattsson-Carlgren et al evaluated the predictive utility of several plasma biomarkers (p-tau181, p-tau217, p-tau231, GFAP, and NfL) in the BioFINDER-1 and the Wisconsin Registry for Alzheimer Prevention (WRAP) study (Mattsson-Carlgren et al, 2023). The combined analysis included 171 cognitively unimpaired but Aβ-positive individuals, with a median follow-up of 6 years and a median age of 73.0 (SD 5.4) and 64.4 years (SD 4.6) in the BioFINDER-1 and WRAP cohorts, respectively. In both cohorts, p-tau217 demonstrated the highest discriminative performance for predicting future cognitive decline among all biomarkers assessed. Conversion from cognitively unimpaired status to AD was evaluated in the BioFINDER-1 cohort only (*n* = 118), where p-tau217 was significantly associated with increased risk of conversion, yielding an HR per 1 SD of log-transformed p-tau217 levels of 2.03 (95% CI: 1.57–2.63) in adjusted regression models. Results for other biomarkers in relation to this outcome were not reported.

### Interpretation of findings

P-tau217 is a marker of tau-phosphorylation associated with cortical fibrillar Aβ plaque deposition, which are both hallmarks of AD pathology (Lane et al, 2018; Shin et al, 2025; Teunissen et al, 2022). In this prospective cohort study, Cox regression analyses revealed associations of p-tau217 with the outcome of AD, with stronger associations in the prodromal (0–9 years) compared to the preclinical phase (9–17 years). Associations of p-tau217 with all-cause dementia were only modest during the first 9 years and lost statistical significance in the preclinical stage in the multivariate model, indicating a specificity of p-tau217 for clinical AD only, as previously indicated in the literature (Ashton et al, 2024; Khalafi et al, 2025; Yu et al, 2023; Gonzalez-Ortiz et al, 2023; Palmqvist et al, 2020). This is reinforced by our study, which, unlike previous cohort studies with a comprehensive set of blood-based biomarker measurements, includes dementia subtypes like VD and MD as separate outcomes, and found no significant associations and a comparably low discriminative ability of p-tau217 with either subtype. On the contrary, but in accordance with previous studies (Stocker et al, 2023a; Baiardi et al, 2022), NfL and GFAP were associated with other dementia subtypes, yielding significantly better discriminative performances of NfL for MD and of GFAP for VD and MD in our study, besides an overall comparable performance to p-tau217 for the outcome of AD. Nevertheless, limitations due to the missing subclassification of all-cause dementia cases need to be acknowledged. Though, sensitivity analyses excluding MD cases yielded consistent results, supporting the robustness of our findings (Appendix Table S5).

Despite similarly good performance of p-tau217, Nfl and GFAP for AD prediction, the prediction accuracy improved to a statistically significant extent when all three markers were combined. This is in line with reports from previous studies and reflects the multi-etiology mostly underlying the pathogenetic processes in later-life cognitive disorders (Grande et al, 2025; Cullen et al, 2021; Teunissen et al, 2022; Gouilly et al, 2023).

A sensitivity analysis revealed that p-tau181 did not contribute to the overall discriminative ability of the biomarker panel, as its exclusion did not yield any changes in the AUC. In Cox regression analyses, p-tau181 was significantly associated with AD in the crude model but narrowly missed statistical significance in the first 9 years of follow-up after adjustment for confounders. These findings align with those of our previous study by Stocker et al and Beyer et al, suggesting p-tau181 as a relatively poor marker for AD (Stocker et al, 2023a; Beyer et al, 2023).

The results regarding the Aβ misfolding biomarker correspond with those from previous studies evaluating this biomarker in the ESTHER study (Nabers et al, 2018; Beyer et al, 2023; Stocker et al, 2020). In the study by Nabers et al, including 65 AD cases among 312 participants, plasma Aβ misfolding predicted AD 14 years before diagnosis with an AUC of 0.80 (95% CI: 0.76–0.84) (Nabers et al, 2018). In a subsequent analysis by Beyer et al, combining Aβ misfolding with GFAP and *APOE* ε4 carrier status improved disease prediction accuracy to an AUC of 0.835 (0.772–0.897) up to 17 years before diagnosis; Aβ alone reached an AUC of 0.784 (0.713–0.854) (Beyer et al, 2023). The performance of the misfolding biomarker for AD risk prediction was even higher in the preclinical stage than in the prodromal stage in an exploratory subgroup analysis of this study. Together, these findings may indicate that Aβ misfolding represents a particularly suitable preclinical indicator of increased AD risk as it captures pathological changes during the preclinical, symptom-free stage up to 17 years before diagnosis. This extended temporal window may provide a critical opportunity for prevention by lifestyle change and possible therapeutic intervention before the onset of clinical symptoms and before substantial, potentially irreversible brain damage has occurred. In contrast, p-tau217 shows similarly strong discriminative performance close to the clinical stage at which large brain damage has occurred. Different biomarkers may be optimally suited to distinct states along the disease trajectory. A major challenge remains that many candidate biomarkers exhibit limited diagnostic utility in the preclinical phase, highlighting the importance of a misfolding biomarker capable of detecting disease-related processes at the preclinical stage.

### Clinical relevance of the findings

In this population-based cohort study, we demonstrated that a combination of three blood-based biomarkers (p-tau217, NfL, and GFAP) with basic demographic and genetic information (age, sex, BMI, and APOE ε4 carrier status) can accurately discriminate future AD diagnosis in the prodromal stage. As this predictive model relies solely on a blood sample and readily available patient data, as well as APOE genotyping, it may offer a cost-effective and scalable tool for routine clinical settings to support risk stratification and etiological assessment in individuals with cognitive impairment or subjective cognitive decline. Such an approach could help identify individuals who may benefit from further confirmatory and cost-intensive diagnostic measures such as PET imaging. Given the nested case-control design of our study, further validation in prospective clinical settings is needed before considering broader implementation.

### Prevention

Given the limitations of current disease-modifying therapies for AD, which are associated with large side effects, the role of blood-based biomarkers in preclinical stages is becoming increasingly important. The therapy response might be more effective, and side effects smaller, if initiated during the preclinical stage, when the amyloid burden is lower. Based on the results from the subgroup analysis, the Aβ misfolding biomarker showed promising performance for the identification of AD risk during the preclinical stage, before significant brain damage occurs. Its predictive accuracy was further improved by incorporating other blood-based biomarkers, reinforcing the multi-etiological nature of late-life cognitive disorders. However, further validation is warranted before adopting this in broader screening applications.

### Strengths and limitations

This study has several strengths, including its prospective cohort design, a comprehensive range of measurements of four prominent blood-based biomarkers, good representation of the German outpatient healthcare setting, and an extensive follow-up period of 17 years.

Nevertheless, some limitations should be acknowledged, including the risk of under- or misdiagnosis of dementia and dementia subtypes due to the community-based nature of the ESTHER study. Dementia diagnoses within the study were made by diverse GPs and medical specialists using varying diagnostic approaches, limiting the ability to verify diagnoses based on cognitive assessments and biomarker status. In addition, dementia subtypes were often not specified or might include misclassification regarding the amyloid status of participants. While this reflects typical conditions in routine outpatient care and enhances the generalizability of the findings, it may partly account for the relatively low number of recorded AD cases. Diagnostic variability and inaccuracies could contribute to an underestimation of associations and may influence the robustness of disease prediction. Also, the outcome of mild cognitive impairment (MCI), which is the primary target group for interventions, was not included in this study. Furthermore, due to resource limitations, p-tau217 measurements could only be performed in baseline blood samples and were not repeated in follow-up samples. Likewise, Aβ misfolding could only be analysed in a subsample of the ESTHER study, limiting the statistical power of the analyses and the precision of the AUC estimates. A detailed comparison between biomarkers should thus be performed based on a larger sample. Lastly, due to the primarily European descent of our study population and the age range of 50 to 75 years at baseline, results may not be generalized to other ethnicities or age groups.

## Conclusion

In this prospective cohort study, plasma p-tau217 proved to be a robust biomarker for clinical AD, showing strong associations and high discriminative accuracy outperforming p-tau181, particularly close to the clinical state. Combining p-tau217 with NfL, GFAP, and basic demographic and genetic factors further improved performance, and reflected the underlying multi-etiology of the disease. These findings highlight the potential of p-tau217 as a central blood-based biomarker for clinical AD. However, the predictive value of p-tau217 was limited in preclinical stages. In contrast, the Aβ misfolding biomarker indicated superior performance as a risk marker in the preclinical phase in an exploratory subgroup analysis. Further longitudinal studies are needed to elucidate the predictive roles of both biomarkers in more depth.

## Methods

**Table:** Reagents and tools table

Reagent/resource	Reference or source	Identifier or catalog number
**Experimental models**		
	N/A	
**Recombinant DNA**		
	N/A	
**Antibodies**		
**Oligonucleotides and other sequence-based reagents**		
	N/A	
**Chemicals, enzymes and other reagents**		
ALZpath Simoa® pTau-217 Advantage PLUS Assay Kit	Quanterix	Ref 104570
**Software**		
Statistical Analysis System (SAS), version 9.4,	https://www.sas.com SAS (Cary, North Carolina, USA)	
**Other**		
Simoa^TM^ HD-X Analyzer by Quanterix^TM^	Quanterix	Simoa™ HD-X Analyzer

### Study design and study population

The ESTHER study (Epidemiologische Studie zu Chancen der Verhütung, Früherkennung und optimierten Therapie chronischer Erkrankungen in der älteren Bevölkerung) is a prospective cohort study initiated in 2000 in Saarland, a federal state in Germany. The study comprises 9940 participants, recruited at baseline (2000–2002) during a general health checkup at their general practitioners (GPs). Besides an age of 50–75 years, the inclusion criteria for the ESTHER study were physical and mental ability to participate in the study, as well as knowledge of the German language. Follow-up of participants took place 2, 5, 8, 11, 14, and 17 years after baseline. For details, see (Stocker et al, 2023b; Trares et al, 2022). The sociodemographic baseline characteristics showed a similar distribution across age categories to those observed in a German National Health Survey conducted with a representative sample of the German population during the recruitment period. The study was conducted in accordance with the Declaration of Helsinki and approved by the ethics committees of the Medical Faculty of the University of Heidelberg (registration number: S-058/2000) and the Physicians’ Board of Saarland, Germany (registration number: 67-00).

### Dementia ascertainment

Information regarding dementia diagnoses was gathered during the 14- and 17-year follow-up of the ESTHER study. In brief, standardized questionnaires were completed by the GPs of study participants, who were asked if they were aware of a dementia diagnosis (including subtypes) among their patients. If so, they were further asked to provide all medical records from neurologists, psychiatrists, memory clinics, or other specialized providers. Dementia information was also collected if participants had already withdrawn from the study due to poor health or death.

### Covariate assessment

Data on age, sex, education, body mass index (BMI), physical activity, smoking status, alcohol consumption, and life-time history of depression were assessed via self-administered questionnaires during baseline assessment. Participants were categorized as having cardiovascular disease (CVD) if their GP reported a diagnosis of CVD, or if a personal history of myocardial infarction, stroke, pulmonary embolism, or coronary artery revascularization was reported in self-administered questionnaires. The history of diabetes was based on GP-reported diagnoses and complemented by GP reports of anti-diabetic drug use.

Kidney function was determined using the estimated glomerular filtration rate test (eGFR) from urine samples taken at baseline. Specifically, the 2021-CKD-EPI creatinine (eGFRcr) equation, excluding race, was used (Inker et al, 2021). Details are described elsewhere (Stocker et al, 2023b).

APOE genotypes were identified using TaqMan single-nucleotide polymorphism (SNP) genotyping assays from Applied Biosystems, California, USA. Endpoint allelic discrimination readings were used to analyze genotypes, utilizing the Bio-Rad CFX Connect System from Bio-Rad Laboratories, CA, USA. If genotyped APOE data were not available (*n* = 70), imputed, quality-controlled data were utilized. For details, see (Stocker et al, 2021).

### Biomarker measurements

P-tau217, p-tau181, GFAP, and NfL levels were measured from baseline EDTA samples. Blood samples were collected by the study participants' GPs and sent to the study center, where they were stored at −80°C until measurement. In preparation for the measurements, samples were thawed at room temperature and centrifuged at 10,000×*g* for 5 min. Subsequently, samples were applied to a canonical 96-well plate (Quanterix, USA) and measured using the Simoa^TM^ HD-X Analyzer by Quanterix^TM^. Concentrations were calculated using lot specific calibrators enclosed with the kits as well as a low and a high concentrated lot specific control. Commercially available Simoa^TM^ Neurology 4-Plex E and 2-plex Advantage Kit (Quanterix, USA), Simoa^TM^ pTau-181 Advantage V2 Kit (Quanterix, USA), and ALZpath Simoa® pTau-217 Advantage PLUS Assay kit were used according to the manufacturer´s instructions and with on-board automated 4x or 3x sample dilution depending on the kit.

Inter-assay variability was assessed using control samples across independent runs (*n* = 11 per level), yielding coefficients of variation (% CV) of 12.2% (low control) and 14.9% (high control), indicating good reproducibility. Intra-assay variability could not be estimated due to single measurements per run. The limit of detection (LOD) was 0.0008 pg/ml (range: 0.0003–0.0021 pg/ml), and values below the LOD were excluded from analyses.

Aβ misfolding was determined using immuno-infrared sensor technology. The method has been reported in detail elsewhere (Nabers et al, 2019; Nabers et al, 2016; Nabers et al, 2018). In summary, this sensor technology detects changes in secondary protein structure and is therefore well suited to assess aggregation and therefore misfolding of proteins such as Aβ, tau, and TAR-DNA binding protein 43 (TDP-43) (Nabers et al, 2019; Nabers et al, 2016; Nabers et al, 2018; Stockmann et al, 2020; Beyer et al, 2021). In plasma, the Aβ misfolding signal reflects the relative abundance of β-sheet-rich, misfolded, and otherwise pathological Aβ conformers in comparison with monomeric, non-toxic forms. The distribution of secondary structures is captured within the amide I spectral region, which serves as an indicator of Aβ misfolding and increases as the disease progresses. For each measurement, the complete Aβ fraction is isolated, and infrared signals are reported in wavenumbers (cm^-1^). Lower wavenumber values correspond to a higher likelihood of AD. Based on prior work, we predefined a cut-off of ≤1642 cm^−1^ to mark this biomarker-associated transition (Nabers et al, 2018). All samples were processed on newly prepared sensor surfaces. About 50 μl of lithium heparin plasma was used per measurement. All analyses were performed under blinded conditions.

### Study sample

This study is based on a nested case-control sample from the ESTHER study, consisting of 513 incident dementia cases and 513 randomly selected controls (Fig. 1), which were selected for measurement of p-tau181, NfL, and GFAP in baseline blood samples in a previous study by Stocker et al (Stocker et al, 2023a). For the current study, complementary measurements of p-tau217, which had not been available at the time of our previous study, were performed in baseline blood samples. However, a small number of samples with insufficient leftover volume (*n* = 6) were excluded from further analysis. Hence, p-tau217 measurements were conducted in baseline blood samples of 1020 ESTHER study participants. Due to a technical application failure in 230 samples and because p-tau217 concentration could not be quantified in 11 samples, the final sample comprised 779 study participants, including 385 incident dementia cases and 394 random controls, which were initially randomly selected from the total cohort. A comparison of baseline characteristics, dementia incidences and previous biomarker measurements (i.e., p-tau181, NfL, and GFAF) showed comparable results (Appendix Table S6), supporting the absence of selection bias. Nevertheless, some influence on effect estimates, particularly for long-term prediction metrics such as HRs and AUC estimates, cannot be completely excluded.

A subgroup of this sample, comprising 55 AD cases and 180 controls, overlapped with the Aβ misfolding biomarker measurements of a previous nested case-control study of the ESTHER study, including 70 AD cases and 707 controls frequency matched on age, sex, and educational level (Nabers et al, 2018; Beyer et al, 2023).

### Statistical analyses

Descriptive statistics were calculated separately for incident all-cause dementia, AD, vascular dementia (VD), and mixed dementia (MD) cases, as well as for controls. Blood-based biomarker levels (p-tau217, p-tau181, NfL, and GFAP) were tested for skewed distribution using the Shapiro–Wilk test. Since the data were not normally distributed, biomarker levels were log-transformed. Outliers were defined as >4 SDs from the mean for each of the blood-based biomarkers, in accordance with Stocker et al (Stocker et al, 2023a), and excluded from further analyses (*n* = 18). Covariates were selected based on a knowledge-based approach.

Cox proportional hazard regression models with various levels of adjustment (model 1—raw model; model 2—adjusted for age, sex, education, and APOE ε4 carrier status; model 3—multivariate model including all covariates: age, sex, education, BMI, physical activity, diabetes, CVD risk, smoking, alcohol consumption, life-time history of depression, kidney function, and *APOE* ε4 carrier status) were used to assess the association between blood-based biomarker levels and incident dementia cases for the full length of follow-up (0–17 years) as well as stratified by time of diagnosis (0–9 and 9–17 years). Hazard ratios (HRs) were computed per 1 standard deviation (SD) increase of the log-transformed biomarker levels. Statistical significance was examined by the Wald test. To assess the robustness of the Cox regression models, sensitivity analyses were performed using logistic regression for the association of p-tau217 with AD at all time-points, showing consistent results (Appendix Table S7). Additionally, for AD analyses, individuals with other or unspecified dementia types (e.g., VD) were excluded. Likewise, VD models excluded cases of AD and other non-vascular dementias, while mixed dementia models excluded cases with only one dementia diagnosis. In addition, the area under the receiver operating characteristic (ROC) curves (AUCs) were computed to explore the discriminative ability of the blood-based biomarkers regarding incident all-cause dementia, AD, VD, and MD diagnoses occurring between baseline and 17 years of follow-up, as well as stratified for time of diagnosis according to previous analyses conducted by Stocker et al (Stocker et al, 2023a) (0–9 and 9–17 years). The comparison of the discriminative ability of the biomarkers was performed using ROC contrast analyses, tested for statistical significance using the DeLong test (DeLong et al, 1988). The dose-response relationship between p-tau217 levels and all four dementia outcomes was assessed using restricted cubic splines (RCS).

All statistical analyses were performed using the Statistical Analysis System (SAS, version 9.4, Cary, North Carolina, USA). Two-sided tests were applied using an alpha level of 0.05.

## Supplementary information

Appendix

Peer Review File

Source data Fig. 2

Source data Fig. 3

Source data Fig. 4

Source data Fig. 5

Source data Fig. 6

Source data Fig. 7

## Data Availability

This study includes no data deposited in external repositories. The source data of this paper are collected in the following database record: biostudies:S-SCDT-10_1038-S44321-026-00483-9.
