# Supplementary material for: Predictive value of plasma p-tau217 for Alzheimer’s dementia compared to other blood-based biomarkers
Source: EMBO Mol Med. 2026 Jul 16;18(8):3374–88. doi: 10.1038/s44321-026-00483-9 (PMC13470377; doi:10.1038/s44321-026-00483-9)
Supplement: Supplementary file 1 [file 44321_2026_483_MOESM1_ESM.pdf]

# Appendix of manuscript entitled

# “Predictive value of plasma p-tau217 for Alzheimer’s dementia compared to other blood-based biomarkers“

## Table of Contents


Appendix Table S1 .......... 2
Appendix Table S2. .......... 4
Appendix Table S3 .......... 5
Appendix Table S4 .......... 6
Appendix Table S5. .......... 7
Appendix Table S6. .......... 8
Appendix Table S7. .......... 10
Appendix Figure S1 .......... 11
Appendix Figure S2 .......... 11
Appendix Figure S3 .......... 11
Appendix Figure S4 .......... 11
Appendix Figure S5 .......... 11

**Appendix Table S1.** Baseline characteristics of included study participants

| Baseline characteristics | Controls (n=394) | Vascular dementia (n=146) | Mixed dementia (n=66) |
|---|---|---|---|
| **Age (years), mean (SD)** | 61.4 (6.5) | 67.4 (4.9) | 67.8 (4.6) |
| **Sex, n (%)** | | | |
| Female | 213 (54.1) | 75 (51.4) | 30 (45.5) |
| Male | 181 (45.9) | 71 (48.6) | 36 (54.5) |
| **Education** | | | |
| ≤9 | 309 (80.3) | 113 (80.7) | 49 (77.8) |
| 10-11 | 39 (10.1) | 15 (10.7) | 7 (1.1) |
| ≥12 | 37 (9.6) | 12 (8.6) | 7 (1.1) |
| **BMI ($kg/m^2$)** | | | |
| <25 | 97 (24.7) | 40 (27.4) | 19 (28.8) |
| 25-30 | 183 (46.7) | 69 (47.3) | 26 (39.4) |
| ≥30 | 112 (28.6) | 37 (25.3) | 21 (31.8) |
| **Physical activity[a]** | | | |
| Inactive | 71 (18) | 43 (29.5) | 24 (36.4) |
| Low | 176 (44.7) | 64 (43.8) | 30 (45.4) |
| Medium or high | 147 (37.3) | 39 (26.7) | 12 (18.2) |
| **Diabetes** | | | |
| No | 338 (86.2) | 109 (74.7) | 48 (73.9) |
| Yes | 54 (13.8) | 37 (25.3) | 17 (26.1) |
| **CVD risk[b]** | | | |
| No | 312 (79.2) | 92 (63.0) | 42 (63.6) |
| Yes | 82 (20.8) | 54 (37.0) | 24 (36.4) |
| **Smoking status** | | | |
| Never smoker | 199 (51.2) | 81 (57.0) | 36 (55.4) |
| Former smoker | 128 (32.9) | 46 (32.4) | 21 (32.3) |
| Current smoker | 62 (15.9) | 15 (10.6) | 8 (12.3) |
| **Alcohol consumption[c]** | | | |
| None | 118 (31.7) | 40 (31.8) | 19 (35.2) |
| Low or moderate | 221 (59.4) | 80 (63.5) | 29 (53.7) |
| High | 22 (8.9) | 6 (4.8) | 6 (1.1) |
| **Life-time history of depression** | | | |
| No | 338 (85.8) | 120 (82.2) | 59 (89.4) |
| Yes | 56 (14.2) | 26 (17.8) | 7 (10.6) |
| **Kidney function** | | | |
| eGFRcr-cys, mean (SD), $mL/min/1.73m^2$ | 83.8 (16.1) | 79.6 (15.9) | 78.8 (15.5) |
| eGFRcr-cys, $mL/min/1.73^2$ | | | |
| ≥90 | 139 (35.3) | 43 (29.5) | 13 (19.7) |
| 60-89 | 222 (56.4) | 86 (58.9) | 47 (71.2) |
| <60 | 33 (8.4) | 17 (11.6) | 6 (9.1) |
| ***APOE* ε4 carrier status** | | | |
| Non-carrier | 282 (73.3) | 88 (65.2) | 45 (69.2) |
| Carrier | 103 (26.8) | 47 (34.8) | 20 (30.8) |

Abbreviations: SD, standard deviation; BMI, body mass index; CVD, cardiovascular disease; *APOE*, apolipoprotein E

[a] "Inactive" was defined by <1h of vigorous or <1h light physical activity per week. "Medium or high" was defined by ≥2h of vigorous and ≥2h of light physical acivity per week. All other amounts of physical activity were grouped into the category "Low"
[b] CVD was defined as diagnosed coronary heart disease or self-reported history of myocardial infarction, stroke, pulmonary embolism, or coronary artery revascularization
[c] Low or moderate alcohol consumption was defined by 0 to 19.99 g ethanol/day (g/d) for women and 0 to 39.99 g/d for men; high alcohol consumption was defined by ≥20 to 39.99 g/d for women and ≥40 g/d for men;

**Appendix Table S2.** Delta AUC for discriminative performance of p-tau217 for the outcome of AD stratified by time of diagnosis

| | | $n_{total}$ | $n_{cases}$ | **AUC (95% CI)** | **ΔAUC (95% CI)** |
|---|---|---|---|---|---|
| 0-9 years | Baseline model[a] | 415 | 39 | 0.863 (0.808-0.919) | - |
| | Extended model[b] | | | 0.894 (0.848-0.940) | 0.031 (-0.005-0.066) |
| 9-17 years | Baseline model[a] | 448 | 72 | 0.765 (0.706-0.823) | - |
| | Extended model[b] | | | 0.780 (0.724-0.836) | 0.015 (-0.007-0.037) |
| 0-17 years | Baseline model[a] | 487 | 111 | 0.787 (0.741-0.833) | - |
| | Extended model[b] | | | 0.811 (0.768-0.854) | 0.024 (0.003-0.440) |

Abbreviations: AUC, Area under the Receiver Operating Curve; CI, confidence interval

[a]The baseline model includes GFAP, NfL, age, sex, BMI, and *APOE ε4* carrier status. Biomarker levels are natural log-transformed levels from baseline.
[b]The extended model includes GFAP, NfL, age, sex, BIM, APOE ε4 carrier status, and p-tau217. Biomarker levels are natural log-transformed levels from baseline.

**Appendix Table S3**. Cox Regression results. HRs for incident **vascular dementia** diagnosis stratified by time of diagnosis.

| | Model 1[a] | | | | Model 2[b] | | | | Model 3[c] | | | |
|---|---|---|---|---|---|---|---|---|---|---|---|---|
| | $n_{total}$ | $n_{cases}$ | HR (95% CI) | P-value[d] | $n_{total}$ | $n_{cases}$ | HR (95% CI) | P-value[d] | $n_{total}$ | $n_{cases}$ | HR (95% CI) | P-value[d] |
| **0-9 years** | | | | | | | | | | | | |
| p-tau217 | 434 | 44 | 1.49 (0.94-2.36) | 0.0924 | 409 | 36 | 0.88 (0.51-1.51) | 0.6387 | 376 | 31 | 1.19 (0.72-1.95) | 0.4987 |
| p-tau181 | 434 | 44 | 1.32 (0.89-1.94) | 0.1654 | 409 | 36 | 0.80 (0.51-1.26) | 0.3384 | 376 | 31 | 0.76 (0.39-1.47) | 0.4069 |
| NfL | 431 | 44 | **2.07 (1.59-2.71)** | **<0.0001** | 406 | 36 | 1.29 (0.87-1.92) | 0.2065 | 373 | 31 | 1.41 (0.84-2.39) | 0.1958 |
| GFAP | 431 | 44 | **2.88 (1.99-4.19)** | **<0.0001** | 406 | 36 | **1.95 (1.14-3.36)** | **0.0156** | 373 | 31 | **2.71 (1.44-5.09)** | **0.0019** |
| **9-17 years** | | | | | | | | | | | | |
| p-tau217 | 486 | 96 | 1.22 (0.88-1.67) | 0.2306 | 460 | 87 | 1.03 (0.71-1.49) | 0.8990 | 424 | 79 | 0.92 (0.63-1.35) | 0.6839 |
| p-tau181 | 486 | 96 | 0.94 (0.69-1.29) | 0.6967 | 460 | 87 | 0.82 (0.63-1.07) | 0.1494 | 424 | 79 | **0.73 (0.56-0.94)** | **0.0141** |
| NfL | 482 | 95 | **1.72 (1.42-2.08)** | **<0.0001** | 456 | 86 | 1.14 (0.86-1.51) | 0.3720 | 420 | 78 | 1.07 (0.79-1.44) | 0.6653 |
| GFAP | 482 | 95 | **1.89 (1.28-2.79)** | **0.0014** | 456 | 86 | 1.28 (0.70-2.34) | 0.4195 | 420 | 78 | 1.39 (0.69-2.81) | 0.3635 |
| **0-17 years** | | | | | | | | | | | | |
| p-tau217 | 530 | 140 | 1.27 (0.99-1.63) | 0.0628 | 496 | 123 | 1.01 (0.78-1.31) | 0.9349 | 455 | 110 | 1.03 (0.80-1.33) | 0.8116 |
| p-tau181 | 530 | 140 | 1.04 (0.82-1.33) | 0.7185 | 496 | 123 | 0.84 (0.67-1.05) | 0.1180 | 455 | 110 | **0.77 (0.62-0.97)** | **0.0268** |
| NfL | 526 | 139 | **1.78 (1.53-2.14)** | **<0.0001** | 492 | 122 | 1.18 (0.93-1.49) | 0.1662 | 451 | 109 | 1.11 (0.86-1.43) | 0.4213 |
| GFAP | 526 | 139 | **2.01 (1.52-2.67)** | **<0.0001** | 492 | 122 | 1.41 (0.91-2.19) | 0.1276 | 451 | 109 | 1.58 (0.95-2.65) | 0.0798 |

Note: Numbers printed in bold are statistically significant (p<.05). All biomarker levels are per 1 SD increase in natural log-transformed biomarker level.
Abbreviations: HR, Hazard Ratio; CI, confidence interval

[a]Model 1: crude model
[b]Model 2: adjusted for age, sex, education, and *APOE* ε4 carrier status
[c]Model 3: multivariable model adjusted for age, sex, education, BMI, physical activity, diabetes, CVD risk, life-time history of depression, kidney function, alcohol consumption, smoking, and *APOE* ε4 carrier status
[d]Results of Wald test

**Appendix Table S4**. Cox Regression results. HRs for incident **mixed dementia** diagnosis stratified by time of diagnosis.

| | **Model 1**[a] | | | | **Model 2**[b] | | | | **Model 3**[c] | | | |
|---|---|---|---|---|---|---|---|---|---|---|---|---|
| | $n_{total}$ | $n_{cases}$ | HR (95% CI) | P-value[d] | $n_{total}$ | $n_{cases}$ | HR (95% CI) | P-value[d] | $n_{total}$ | $n_{cases}$ | HR (95% CI) | P-value[d] |
| **0-9 years** | | | | | | | | | | | | |
| p-tau217 | 407 | 17 | **1.93 (1.03-3.60)** | **0.0393** | 390 | 17 | 1.35 (0.67-2.73) | 0.3998 | 358 | 13 | 1.78 (0.77-4.13) | 0.1802 |
| p-tau181 | 407 | 17 | **1.68 (1.06-2.67)** | **0.0283** | 390 | 17 | 1.32 (0.69-2.52) | 0.4031 | 358 | 13 | **2.82 (1.21-6.55)** | **0.0162** |
| NfL | 404 | 17 | **2.85 (2.02-4.03)** | **<0.0001** | 387 | 17 | **2.28 (1.36-3.83)** | **0.0019** | 355 | 13 | **5.69 (2.93-11.07)** | **<0.0001** |
| GFAP | 404 | 17 | **2.92 (1.55-5.49)** | **0.0009** | 387 | 17 | 1.96 (0.75-5.09) | 0.1675 | 355 | 13 | 3.36 (0.64-17.63) | 0.1524 |
| **9-17 years** | | | | | | | | | | | | |
| p-tau217 | 436 | 46 | **1.59 (1.12-2.25)** | **0.0096** | 415 | 42 | 1.21 (0.82-1.79) | 0.3409 | 380 | 35 | 1.26 (0.80-1.99) | 0.3227 |
| p-tau181 | 436 | 46 | 1.37 (0.95-1.97) | 0.0904 | 415 | 42 | 0.90 (0.62-1.32) | 0.6012 | 380 | 35 | 0.94 (0.53-1.66) | 0.8233 |
| NfL | 433 | 46 | **2.07 (1.58-2.70)** | **<0.0001** | 412 | 42 | 1.10 (0.74-1.63) | 0.6290 | 377 | 35 | 1.08 (0.61-1.91) | 0.8042 |
| GFAP | 433 | 46 | **2.91 (2.02-4.18)** | **<0.0001** | 412 | 42 | **2.02 (1.17-3.49)** | **0.0114** | 377 | 35 | **2.38 (1.19-4.75)** | **0.0138** |
| **0-17 years** | | | | | | | | | | | | |
| p-tau217 | 453 | 63 | **1.65 (1.22-2.24)** | **0.0013** | 432 | 59 | 1.24 (0.86-1.77) | 0.2478 | 393 | 48 | 1.34 (0.89-2.03) | 0.1662 |
| p-tau181 | 453 | 63 | **1.44 (1.07-1.94)** | **0.0158** | 432 | 59 | 0.98 (0.70-1.37) | 0.8954 | 393 | 48 | 1.15 (0.70-1.90) | 0.5844 |
| NfL | 450 | 63 | **2.27 (1.80-2.87)** | **<0.0001** | 429 | 59 | 1.37 (0.97-1.92) | 0.0706 | 390 | 48 | **1.61 (1.03-2.54)** | **0.0386** |
| GFAP | 450 | 63 | **2.77 (2.03-3.79)** | **<0.0001** | 429 | 59 | **1.89 (1.19-3.02)** | **0.0072** | 390 | 48 | **2.30 (1.27-4.15)** | **0.0059** |

Note: Numbers printed in bold are statistically significant (p<.05). All biomarker levels are per 1 SD increase in natural log-transformed biomarker level.
Abbreviations: HR, Hazard Ratio; CI, confidence interval

[a]Model 1: crude model
[b]Model 3: adjusted for age, sex, education, and *APOE* ε4 carrier status
[c]Model 4: multivariable model adjusted for age, sex, education, BMI, physical activity, diabetes, CVD risk, life-time history of depression, kidney function, alcohol consumption, smoking, and *APOE* ε4 carrier status
[d]Results of Wald test

**Appendix Table S5.** Cox Regression results. HRs for incident **vascular dementia** diagnosis **without mixed dementia** diagnoses stratified by time of diagnosis.

| | Model 1[a] | | | | Model 2[b] | | | | Model 3[c] | | | |
|---|---|---|---|---|---|---|---|---|---|---|---|---|
| | $n_{total}$ | $n_{cases}$ | HR (95% CI) | P-value[d] | $n_{total}$ | $n_{cases}$ | HR (95% CI) | P-value[d] | $n_{total}$ | $n_{cases}$ | HR (95% CI) | P-value[d] |
| **0-9 years** | | | | | | | | | | | | |
| p-tau217 | 428 | 38 | 1.52 (0.92-2.52) | 0.1041 | 403 | 30 | 0.86 (0.47-1.59) | 0.6331 | 371 | 26 | 1.10 (0.64-1.87) | 0.7390 |
| **9-17 years** | | | | | | | | | | | | |
| p-tau217 | 474 | 84 | **1.15 (0.81-1.65)** | **0.4171** | 449 | 76 | 0.95 (0.64-1.40) | 0.7885 | 413 | 68 | 0.84 (0.60-1.18) | 0.3183 |
| **0-17 years** | | | | | | | | | | | | |
| p-tau217 | 512 | 122 | 1.24 (0.94-1.64) | 0.1245 | 479 | 106 | 0.97 (0.74-1.26) | 0.7944 | 439 | 94 | 0.96 (0.75-1.22) | 0.7169 |

Note: Numbers printed in bold are statistically significant (p<.05). All biomarker levels are per 1 SD increase in natural log-transformed biomarker level.
Abbreviations: HR, Hazard Ratio; CI, confidence interval

[a]Model 1: crude model
[b]Model 2: adjusted for age, sex, education, and *APOE* ε4 carrier status
[c]Model 3: multivariable model adjusted for age, sex, education, BMI, physical activity, diabetes, CVD risk, life-time history of depression, kidney function, alcohol consumption, smoking, and *APOE* ε4 carrier status
[d]Results of Wald test

**Appendix Table S6.** Baseline characteristics of included (n=779) and excluded (n=241) study participants

| Baseline characteristics | Included study participants | Excluded study participants |
|---|---|---|
| **Age (years), mean (SD)** | 64.2 (6.5) | 63.9 (6.4) |
| **Sex, n (%)** | | |
| Female | 412 (52.9) | 139 (57.7) |
| Male | 367 (47.1) | 102 (42.3) |
| **Education** | | |
| ≤9 | 610 (80.9) | 182 (77.8) |
| 10-11 | 74 (9.8) | 25 (10.7) |
| ≥12 | 70 (9.3) | 27 (11.5) |
| **BMI (kg/m$^2$)** | | |
| <25 | 217 (28) | 59 (24.5) |
| 25-30 | 353 (45.5) | 107 (44.4) |
| ≥30 | 206 (26.6) | 75 (31.1) |
| **Physical activity**[a] | | |
| Inactive | 185 (23.8) | 58 (24.1) |
| Low | 343 (44.1) | 115 (47.7) |
| Medium or high | 250 (32.1) | 68 (28.2) |
| **Diabetes** | | |
| No | 637 (82.4) | 192 (82.1) |
| Yes | 136 (17.6) | 42 (18) |
| **CVD**[b] | | |
| No | 580 (74.5) | 184 (76.4) |
| Yes | 199 (25.6) | 57 (23.7) |
| **Smoking status** | | |
| Never smoker | 409 (53.8) | 116 (49.8) |
| Former smoker | 247 (32.5) | 79 (33.9) |
| Current smoker | 104 (13.7) | 38 (16.3) |
| **Alcohol consumption**[c] | | |
| None | 230 (33.1) | 76 (35.2) |
| Low or moderate | 414 (59.5) | 121 (56.0) |
| High | 52 (7.5) | 19 (8.8) |
| **Life-time history of depression** | | |
| No | 663 (85.1) | 210 (87.1) |
| Yes | 116 (14.9) | 31 (12.9) |
| **Kidney function** | | |
| eGFRcr-cys, mean (SD), mL/min/1.73m$^2$ | 81.8 (16.4) | 81.5 (16.8) |
| eGFRcr-cys, mL/min/1.73$^2$ | | |
| ≥90 | 247 (31.7) | 91 (37.8) |
| 60-89 | 454 (58.3) | 122 (50.6) |
| <60 | 78 (10) | 28 (11.6) |
| ***APOE* ε4 carrier status** | | |
| Non-carrier | 498 (66.7) | 159 (69.4) |
| Carrier | 249 (33.3) | 70 (30.6) |
| **All-cause dementia** | | |
| No | 394 (50.6) | 119 (49.4) |
| Yes | 385 (49.4) | 122 (50.6) |

| Baseline characteristics | Included study participants | Excluded study participants |
|---|---|---|
| **Alzheimer's dementia** | | |
| No | 656 (84.2) | 200 (83.0) |
| Yes | 123 (15.8) | 41 (17.0) |
| **Vascular dementia** | | |
| No | 633 (81.3) | 193 (80.1) |
| Yes | 146 (18.7) | 48 (19.9) |
| **Mixed dementia** | | |
| No | 713 (91.5) | 224 (93.0) |
| Yes | 66 (8.5) | 17 (7.1) |
| **Baseline biomarker plasma levels (pg/ml), mean (SD)** | | |
| p-tau181 | 1.9 (2.3) | 1.8 (1.4) |
| GFAP | 109.1 (65.6) | 105.4 (78.3) |
| NfL | 18.1 (10.7) | 18.1 (12.3) |

Abbreviations: SD, standard deviation; BMI, body mass index; CVD, cardiovascular disease; *APOE*, apolipoprotein E; p-tau181, phosphorylated-tau181; GFAP, glial fibrillary acidic protein; NFL, neurofilament light chain

[a] "Inactive" was defined by <1h of vigorous or <1h light physical activity per week. "Medium or high" was defined by ≥2h of vigorous and ≥2h of light physical activity per week. All other amounts of physical activity were grouped into the category "Low"
[b] CVD was defined as diagnosed coronary heart disease or self-reported history of myocardial infarction, stroke, pulmonary embolism, or coronary artery revascularization
[c] Low or moderate alcohol consumption was defined by 0 to 19.99 g ethanol/day (g/d) for women and 0 to 39.99 g/d for men; high alcohol consumption was defined by ≥20 to 39.99 g/d for women and ≥40 g/d for men;

**Appendix Table S7.** Logistic Regression results. ORs for incident **Alzheimer's dementia** diagnosis stratified by time of diagnosis.

| | Model 1[a] | | | | Model 2[b] | | | | Model 3[c] | | | |
|---|---|---|---|---|---|---|---|---|---|---|---|---|
| | $n_{total}$ | $n_{cases}$ | HR (95% CI) | P-value[d] | $n_{total}$ | $n_{cases}$ | HR (95% CI) | P-value[d] | $n_{total}$ | $n_{cases}$ | HR (95% CI) | P-value[d] |
| **0-9 years** | | | | | | | | | | | | |
| p-tau217 | 432 | 42 | **3.27 (2.23-4.78)** | **<0.0001** | 411 | 38 | **2.54 (1.61-4.00)** | **<0.0001** | 377 | 32 | **3.00 (1.75-5.14)** | **<0.0001** |
| **9-17 years** | | | | | | | | | | | | |
| p-tau217 | 467 | 77 | **1.90 (1.43-2.52)** | **<0.0001** | 444 | 71 | **1.59 (1.16-2.17)** | **0.0038** | 404 | 59 | **1.67 (1.17-2.38)** | **0.0047** |
| **0-17 years** | | | | | | | | | | | | |
| p-tau217 | 509 | 119 | **2.25 (1.77-2.87)** | **<0.0001** | 482 | 109 | **1.81 (1.39-2.37)** | **<0.0001** | 436 | 91 | **1.93 (1.43-2.61)** | **<0.0001** |

Note: Numbers printed in bold are statistically significant (p<.05). All biomarker levels are per 1 SD increase in natural log-transformed biomarker level.
Abbreviations: HR, Hazard Ratio; CI, confidence interval

[a]Model 1: crude model
[b]Model 2: adjusted for age, sex, education, and *APOE* ε4 carrier status
[c]Model 3: multivariable model adjusted for age, sex, education, BMI, physical activity, diabetes, CVD risk, life-time history of depression, kidney function, alcohol consumption, smoking, and *APOE* ε4 carrier status
[d]Results of Wald test

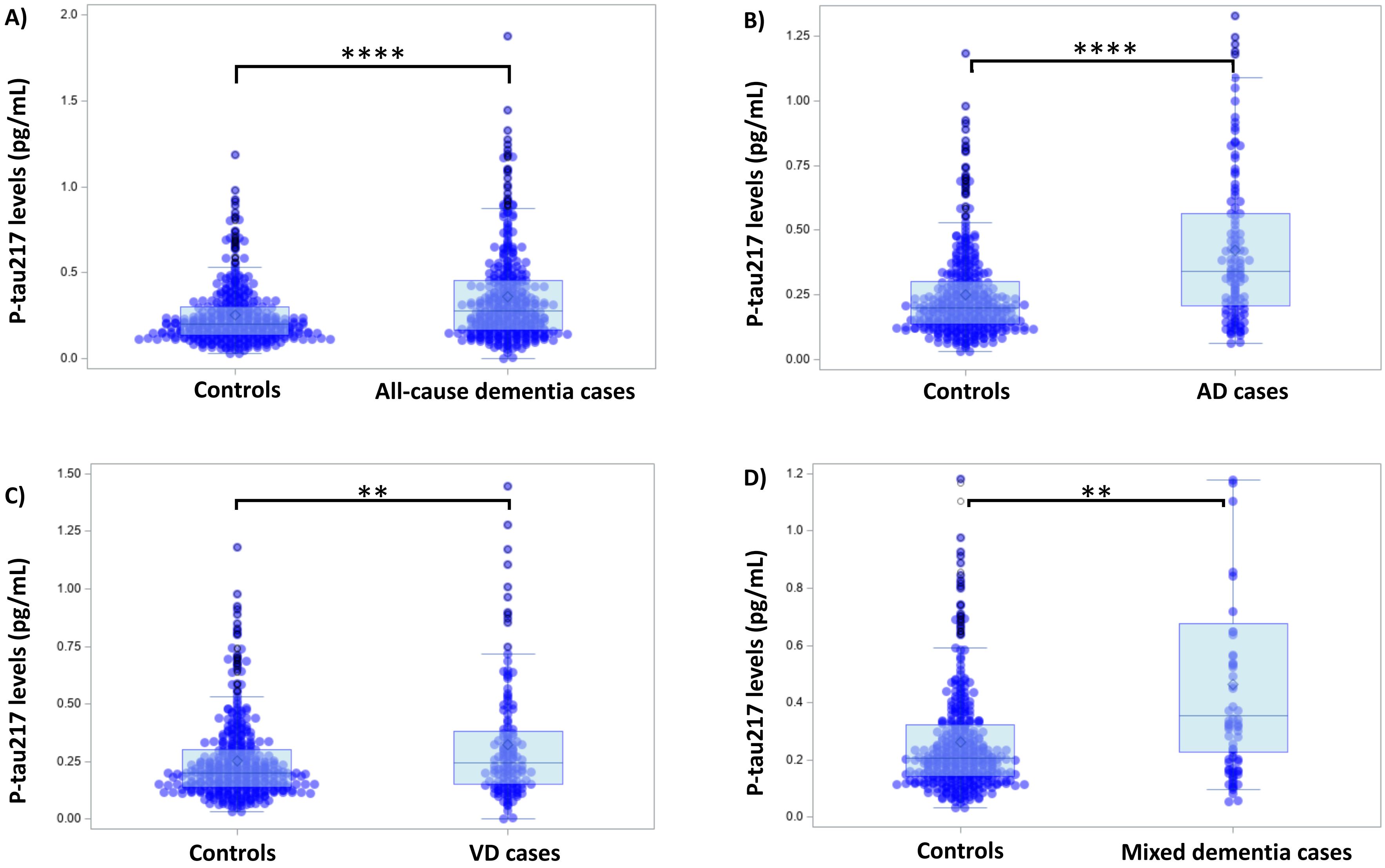


**Appendix Figure S1.** Baseline plasma p-tau217 levels by incident A) all-cause dementia, B) Alzheimer's dementia, c) vascular dementia diagnosis and d) mixed dementia diagnosis compared to controls, respectively. Median (interquartile range (IQR)) p-tau217 levels (pg/ml) for controls: 0.2 (0.1-0.3). *P < .05, **P < .01, ***P < .001, ****P < .0001

**A) p=8.61 × $10^{-10}$;** p-tau217 levels (pg/ml) median (IQR): 0.3 (0.2-0.5); $n_{cases}$=371, $n_{controls}$=390
**B) p=8.88 × $10^{-11}$;** p-tau217 levels (pg/ml) median IQR): 0.3 (0.2-0.6); $n_{cases}$=119, $n_{controls}$=390
**C) p=0.0088;** p-tau217 levels (pg/ml) median (IQR): 0.2 (0.1-0.4); $n_{cases}$=140, $n_{controls}$=390
**D) p=0.0034;** p-tau217 levels (pg/ml) median (IQR): 0.3 (0.2-0.5); $n_{cases}$=63, $n_{controls}$=390

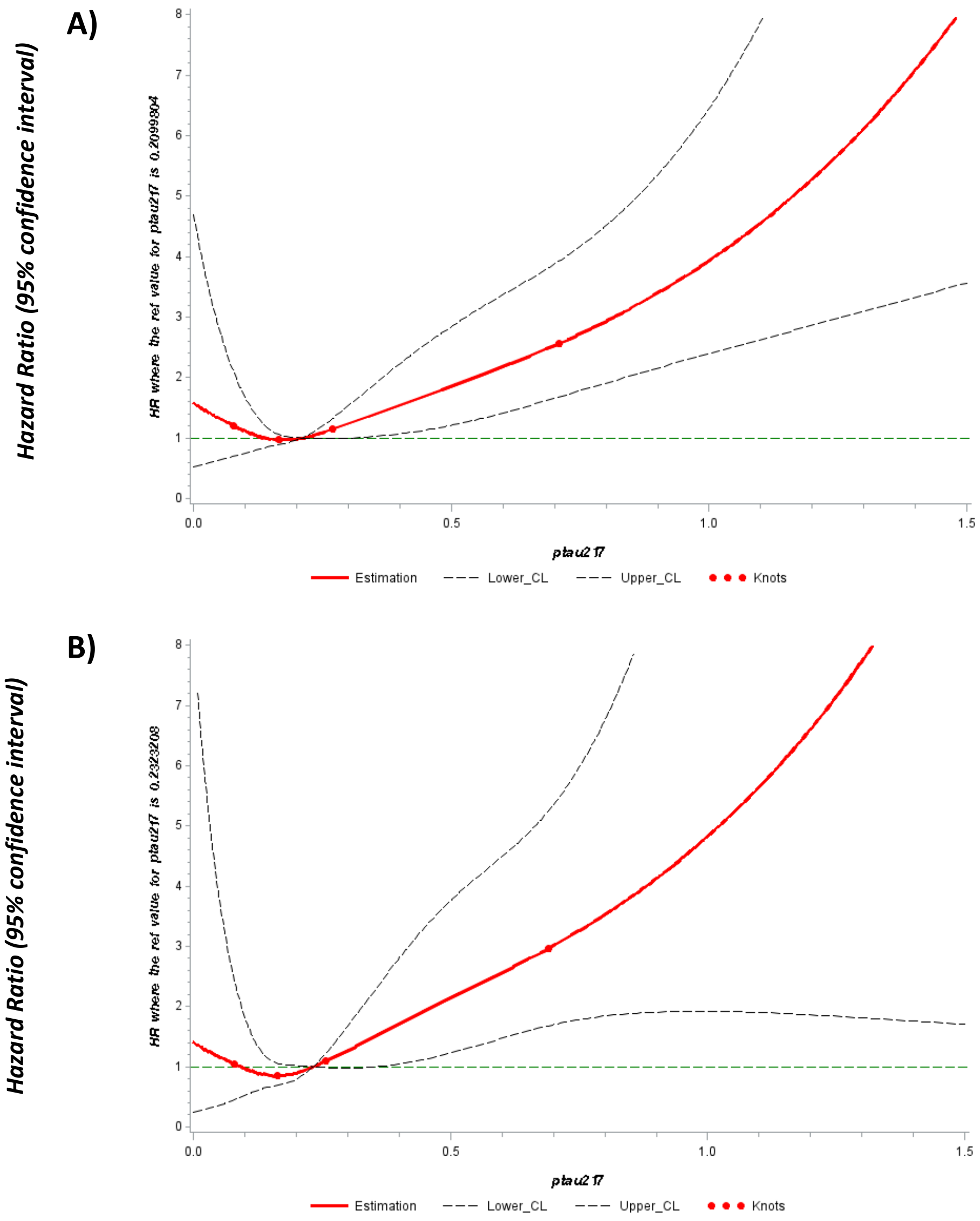


**Appendix Figure S2**. Dose-response relationship between p-tau217 biomarker levels at baseline and incident A) vascular dementia, and B) mixed dementia diagnosis within 17 years in a spline regression model. Knots were placed at the 5th, 35th, 65th and 90th percentiles of p-tau217 levels.

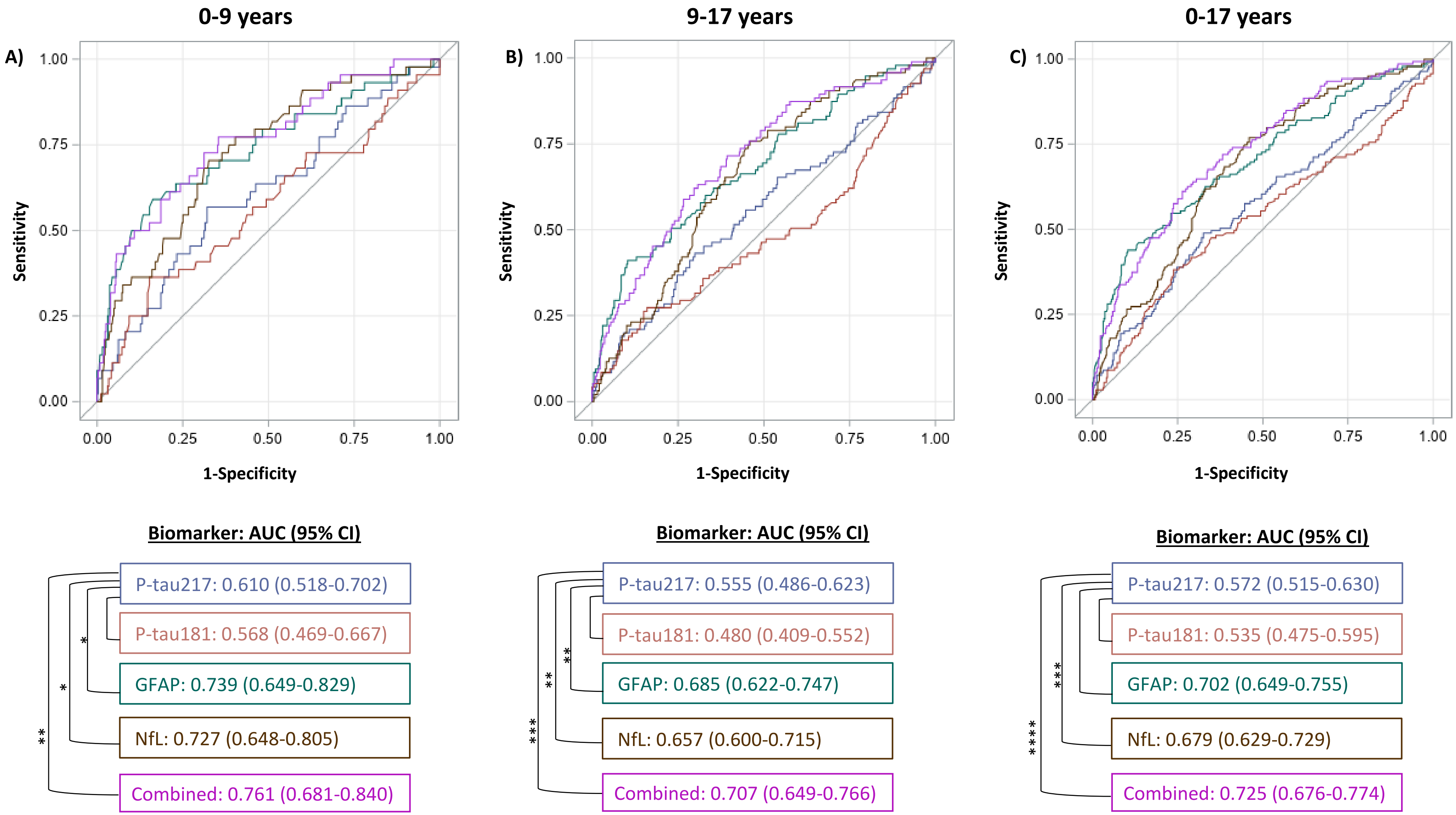


**Appendix Figure S3**. ROC curves and contrast using DeLong test for incident **vascular dementia** within A) 0-9 years, B) 9-17 years and C) 0-17 years of follow-up based on baseline biomarker levels. Biomarker levels are natural log-transformed levels from baseline. **P* < .05, ***P* < .01, ****P* < .001, *****P* < .0001 (DeLong Test).

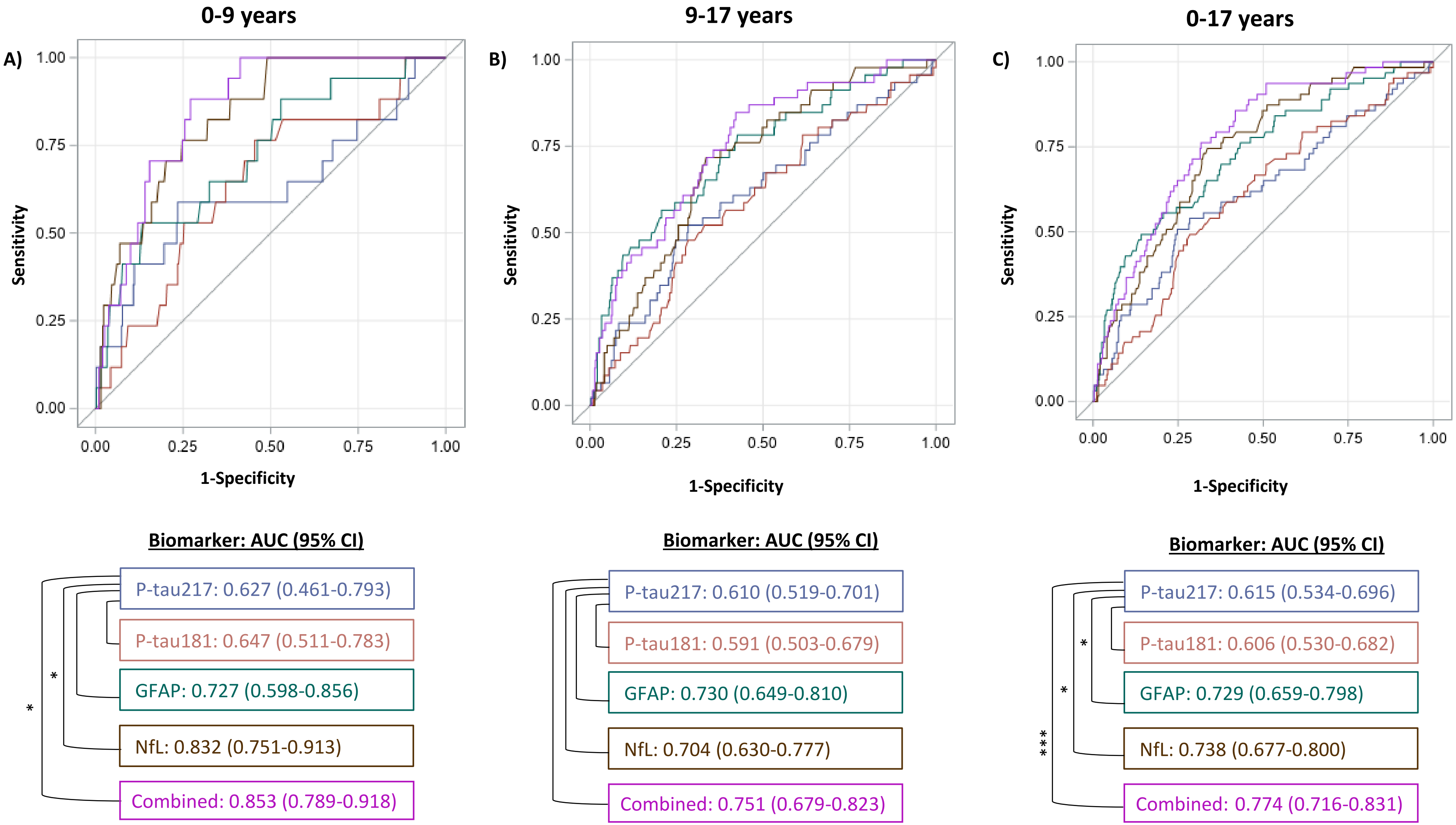


**Appendix Figure S4**. ROC curves and contrast using DeLong test for incident **mixed dementia** within A) 0-9 years, B) 9-17 years and C) 0-17 years of follow-up based on baseline biomarker levels. Biomarker levels are natural log-transformed levels from baseline. **P* < .05, ***P* < .01, ****P* < .001, *****P* < .0001 (DeLong Test).

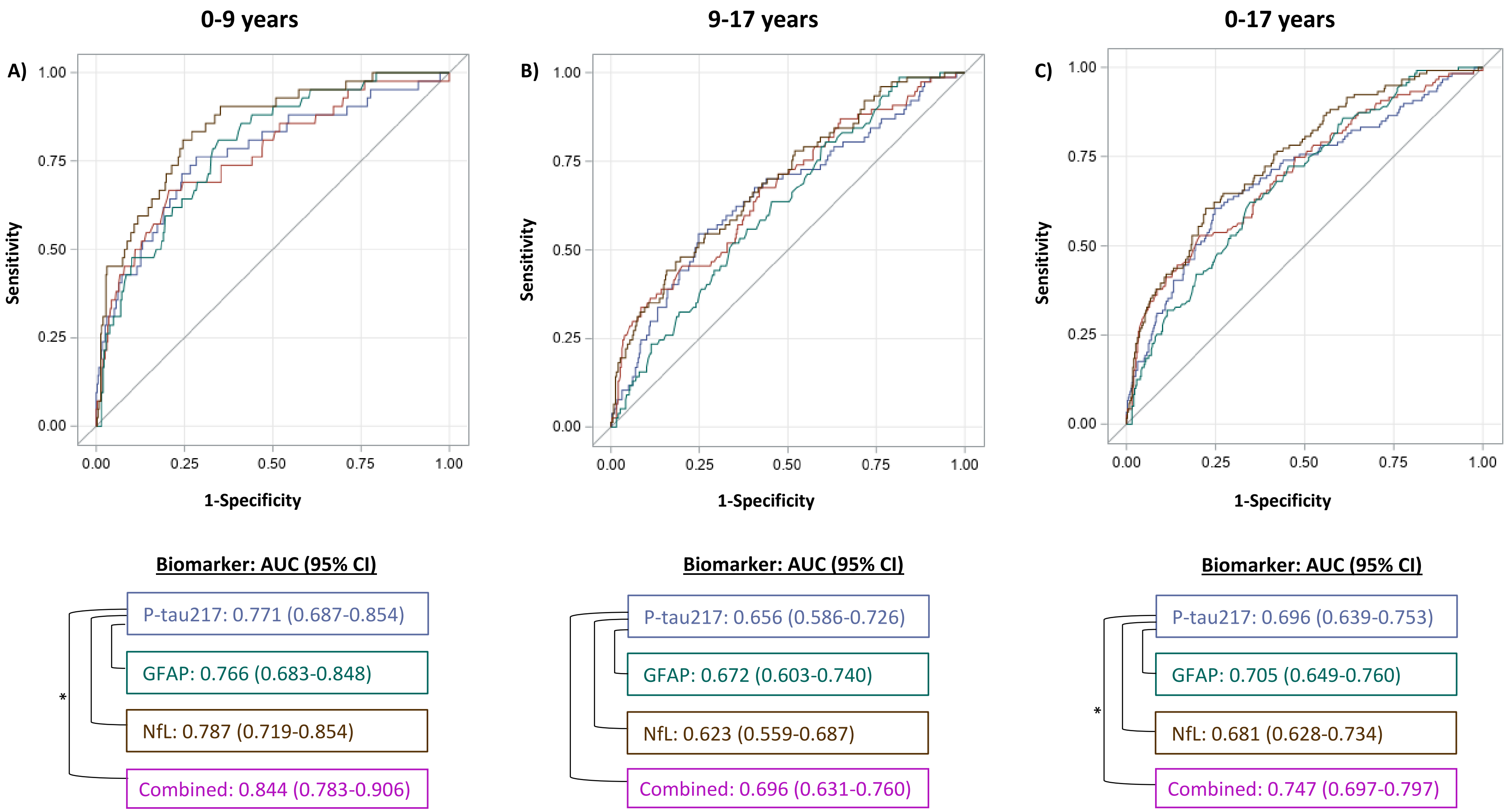


**Appendix Figure S5**. ROC curves and contrast using DeLong test for incident **Alzheimer's dementia** within A) 0-9 years, B) 9-17 years and C) 0-17 years of follow-up based on baseline biomarker levels without p-tau181. Biomarker levels are natural log-transformed levels from baseline. *$P$ < .05, **$P$ < .01, ***$P$ < .001, ****$P$ < .0001 (DeLong Test).
