# Supplementary material for: Predictive value of plasma p-tau217 for Alzheimer’s dementia compared to other blood-based biomarkers
Source: EMBO Mol Med. 2026 Jul 16;18(8):3374–88. doi: 10.1038/s44321-026-00483-9 (PMC13470377; doi:10.1038/s44321-026-00483-9)
Supplement: Supplementary file 2 [file 44321_2026_483_MOESM2_ESM.pdf]

EMBO Molecular Medicine

Predictive value of plasma p-tau217 for Alzheimer's dementia compared to other blood-based biomarkers

Kira Trares, Deniz Duman, Léon Beyer, Johannes Michaelian, Hannah Stocker, Bernd Holleczek, Konrad Beyreuther, Ben Schöttker, Klaus Gerwert, and Hermann Brenner

Corresponding authors: Hermann Brenner (h.brenner@Dkfz-Heidelberg.de)

Review Timeline:

Submission Date:	21st Jan 26
Editorial Decision:	17th Feb 26
Revision Received:	5th May 26
Editorial Decision:	3rd Jun 26
Revision Received:	15th Jun 26
Accepted:	26th Jun 26

Editor: Jingyi Hou

Transaction Report:

(Note: With the exception of the correction of typographical or spelling errors that could be a source of ambiguity, letters and reports are not edited. Depending on transfer agreements, referee reports obtained elsewhere may or may not be included in this compilation. Referee reports are anonymous unless the Referee chooses to sign their reports.)

17th Feb 2026

Dear Prof. Brenner,

Thank you for submitting your work to EMBO Molecular Medicine. We have now received evaluations from the three reviewers who agreed to assess your manuscript. As you will see in the reports below, the referees find your study of interest but have raised several concerns that will need to be addressed in a major revision of the manuscript.

The referees' recommendations are rather clear, so I won't repeat the points listed below. It is important to carefully address all the issues raised by the referees. Please feel free to contact me in case you would like to discuss in further detail any of the issues raised by the reviewers.

We would welcome the submission of a revised version in three months for further consideration. As you may already know, our editorial policy allows in principle a single round of major revision, and it is therefore essential to provide responses to the referees' comments that are as complete as possible.

EMBO Molecular Medicine has a "scooping protection" policy, whereby similar findings that are published by others during review or revision are not a criterion for rejection. Should you decide to submit a revised version, I do ask that you get in touch after three months if you have not completed it, to update us on the status.

Please also contact us as soon as possible if similar work is published elsewhere. If other work is published, we may not be able to extend the revision period beyond three months.

Please read below for important editorial formatting and consult our author's guidelines for proper formatting of your revised article for EMBO Molecular Medicine.

I look forward to receiving your revised manuscript.

Yours sincerely,
Jingyi

Jingyi Hou
Senior Editor
EMBO Molecular Medicine

*****
When preparing your revised manuscript, please refer to our guidelines: https://link.springer.com/journal/44321/submission-guidelines#cms-Revised-submissions. We perform an initial quality control of all revised manuscripts before re-review; failure to include requested items will delay the evaluation of your revision.

We require:

1) A .docx formatted version of the manuscript text (including legends for main figures, EV figures and tables). Please make sure that the changes are highlighted to be clearly visible.

2) Individual production quality figure files as .eps, .tif, .jpg (one file per figure). For guidance, download the 'Figure Guide PDF': https://media.springernature.com/original/springer-cms/rest/v1/content/27825798/data/v1.

3) A .docx formatted letter INCLUDING the reviewers' reports and your detailed point-by-point responses to their comments. As part of the EMBO Press transparent editorial process, the point-by-point response is part of the Review Process File (RPF), which will be published alongside your paper.

4) A complete author checklist, which you can download from our author guidelines. Please insert information in the checklist that is also reflected in the manuscript. The completed author checklist will also be part of the RPF.

5) Please note that all corresponding authors are required to supply an ORCID ID for their name upon submission of a revised manuscript.

6) It is mandatory to include a 'Data Availability' section after the Materials and Methods. Before submitting your revision, primary datasets produced in this study need to be deposited in an appropriate public database, and the accession numbers and database listed under 'Data Availability'. Please remember to provide a reviewer password if the datasets are not yet public.

In case you have no data that requires deposition in a public database, please state so in this section. Note that the Data Availability Section is restricted to new primary data that are part of this study.

7) For data quantification: please specify the name of the statistical test used to generate error bars and P values, the number (n) of independent experiments (specify technical or biological replicates) underlying each data point and the test used to calculate p-values in each figure legend. The figure legends should contain a basic description of n, P and the test applied. Graphs must include a description of the bars and the error bars (s.d., s.e.m.).

8) At EMBO Press we ask authors to provide source data for the main manuscript figures. You will receive a separate email with instructions for providing source data with your revised manuscript, including how to upload and organize the files.

9) Our journal encourages inclusion of *data citations in the reference list* to directly cite datasets that were re-used and obtained from public databases. Data citations in the article text are distinct from normal bibliographical citations and should directly link to the database records from which the data can be accessed. In the main text, data citations are formatted as follows: "Data ref: Smith et al, 2001" or "Data ref: NCBI Sequence Read Archive PRJNA342805, 2017". In the Reference list, data citations must be labeled with "[DATASET]". A data reference must provide the database name, accession number/identifiers and a resolvable link to the landing page from which the data can be accessed at the end of the reference.

10) We replaced Supplementary Information with Expanded View (EV) Figures and Tables that are collapsible/expandable online. A maximum of 5 EV Figures can be typeset. EV Figures should be cited as 'Figure EV1, Figure EV2" etc... in the text and their respective legends should be included in the main text after the legends of regular figures.

- For the figures that you do NOT wish to display as Expanded View figures, they should be bundled together with their legends in a single PDF file called *Appendix*, which should start with a short Table of Content. Appendix figures should be referred to in the main text as: "Appendix Figure S1, Appendix Figure S2" etc.

- Additional Tables/Datasets should be labeled and referred to as Table EV1, Dataset EV1, etc. Legends have to be provided in a separate tab in case of .xls files. Alternatively, the legend can be supplied as a separate text file (README) and zipped together with the Table/Dataset file.

11) The paper explained: EMBO Molecular Medicine articles are accompanied by a summary of the articles to emphasize the major findings in the paper and their medical implications for the non-specialist reader. Please provide a draft summary of your article highlighting

- the medical issue you are addressing,

- the results obtained and

- their clinical impact.

This may be edited to ensure that readers understand the significance and context of the research. Please refer to any of our published articles for an example.

12) Author contributions: You will be asked to provide CRediT (Contributor Role Taxonomy) terms in the submission system. These replace a narrative author contribution section in the manuscript.

13) A Conflict of Interest statement should be provided in the main text.

14) Please provide a 'Synopsis' to further enhance discoverability. Synopses are displayed on the journal webpage and are freely accessible to all readers. They include a short stand first (maximum of 300 characters, including space) as well as 2-5 one-sentences bullet points that summarizes the paper. Please write the bullet points to summarize the key NEW findings. They should be designed to be complementary to the abstract - i.e. not repeat the same text. We encourage inclusion of key acronyms and quantitative information (maximum of 30 words / bullet point). Please use the passive voice. Please attach these in a separate file or send them by email, we will incorporate them accordingly.

Please also provide a visual abstract to illustrate your article as a PNG file 550 px wide x 300-600 px high.

15) Please download and fill our Reagents and Tools Table template (.docx), which you can find in our author guidelines:

https://link.springer.com/journal/44320/submission-guidelines#structuredmethods.

When submitting your revised manuscript, please do not include the Reagents and Tools Table in the Methods section of the manuscript but upload it as a separate file choosing the file type "Reagent Table".

EMBO Molecular Medicine has a "scooping protection" policy, whereby similar findings that are published by others during review or revision are not a criterion for rejection. Should you decide to submit a revised version, I do ask that you get in touch after three months if you have not completed it, to update us on the status.

***** Reviewer's comments *****

Referee #1 (Comments on Novelty/Model System for Author):

This manuscript evaluates the predictive value of plasma p-tau217 for Alzheimer's disease (AD) in a large, population-based prospective cohort with long follow-up, and compares its performance with other blood-based biomarkers (p-tau181, NfL, GFAP), as well as with an amyloid-β (Aβ) misfolding biomarker in a subgroup. The long follow-up (up to 17 years), prospective design, and availability of multiple biomarkers measured at baseline represent major strengths. The work addresses an important and timely question regarding the temporal utility of blood biomarkers across different stages of AD development.
Major comments
A considerable proportion of samples initially selected for p-tau217 measurement were excluded due to technical failure or non-quantifiable concentrations (approximately 24% of samples with available material). The manuscript does not sufficiently address whether excluded participants differed systematically from those included (e.g., age, sex, dementia status, biomarker levels from prior assays). The authors should provide a comparison of baseline characteristics between included and excluded participants, and discuss how assay failure and missingness might bias hazard ratios and AUC estimates, particularly for long-term prediction.
Although the study is based on a nested case-control sample, Cox proportional hazards models are applied without clear justification or weighting. It is unclear whether the sampling strategy preserves valid hazard ratio estimates for the underlying cohort, especially for subtype-specific dementia analyses. The authors should clarify whether and how the nested design was accounted for in the Cox model and justify the use of time-to-event analyses rather than conditional logistic regression, or provide sensitivity analyses demonstrating robustness of results.
The manuscript frequently describes p-tau217 as performing well "early," yet the strongest associations are observed within 0- 9 years prior to diagnosis, which may already correspond to prodromal or near-clinical disease rather than true preclinical AD. In contrast, the Aβ misfolding biomarker appears to perform better beyond 9 years. I suggest refining the conceptual framework distinguishing preclinical, prodromal, and near-clinical phases. Please, avoid overstating p-tau217's utility for "early" risk prediction when its performance declines substantially beyond 9 years.
Comparisons between p-tau217 and Aβ misfolding are based on a small subgroup (55 AD cases, 180 controls). Despite limited power, strong conclusions are drawn regarding the superiority of Aβ misfolding for long-term prediction. Please, emphasize the exploratory nature of these analyses and include wider discussion of uncertainty and potential instability of AUC estimates in small subgroups.
Dementia diagnoses were GP-reported with heterogeneous diagnostic work-ups, and dementia subtypes were often unspecified. This limitation is acknowledged but not sufficiently integrated into the interpretation of subtype-specific findings (VD, MD). Please, discuss more explicitly how misclassification may differentially affect biomarker performance, particularly for p-tau217 specificity, and consider sensitivity analyses excluding mixed or unspecified cases, if feasible.
While combined biomarker models yield high AUCs, it remains unclear how much incremental value p-tau217 adds beyond GFAP, NfL, and demographic/genetic variables. Please, provide formal measures of incremental predictive value (e.g., ΔAUC, net reclassification improvement, or decision-curve analysis) and clarify whether p-tau217 meaningfully improves clinical decision-making rather than statistical performance alone.

Minor comments

- Specify limits of detection and inter-/intra-assay variability for p-tau217.
- Clarify handling of values below detection limits.
- Report proportional hazards assumption testing.
- Justify the choice of 9-year cut-point for follow-up stratification.
- Some figures (ROC curves) are difficult to interpret due to overlapping lines; consider simplifying or moving additional curves to supplementary material
- Ensure consistent terminology when referring to "prediction," "association," and "discrimination."
- Moderate claims suggesting clinical readiness of p-tau217 for population screening, given observed limitations for long-term prediction.

Referee #2 (Remarks for Author):

This study used a nested case-control design in the prospective ESTHER cohort with up to 17 years follow-up to test the predictive accuracy of different biomarkers, including p-tau217, for AD, vascular dementia and mixed dementia. They showed that p-tau217, NfL and GFAP have higher predictive accuracy within the first 9 years of follow-up while an amyloid aggregation assay has a higher predictive accuracy in later follow-ups. All in all, this is an intersting and important study that investigates the use of these biomarkers on a population-level, non-specialized dementia care setting. They use adequate statistical models with covariate corrections. However, I have the following converns, particularly regarding the interpretation of the usability of these biomarkers in cognitively unimpaired individuals. The following analyses should be added to further determine whether the general population should be target of these biomarkers:

- Dementia diagnosis and etiology are based on standardized questionnaires. However, it is not possible to verify this by the cognitive and biomarker status at the time of diagnosis. Also MCI is not included as diagnosis which is the main target group for interventions. These should be included as important limitations.
- Are there any baseline characteristics regarding the cognitive status of the included individuals? It is important to emphasize if they were non-demented for the interpretation of the results.
- Please calculate the positive and negative predictive values for the different time episodes and biomarkers. I do not agree with the conclusion, especially lines 425-427. Likely, the PPV will show that p-tau217 is not suited as a screening tool in people without cognitive deficits. This interpretation would be in line with literature (e.g. PMID: 39533113, 37653254) showing that p-tau217 should be only used in people with MCI in the clinical setting.
- Please also include a power analysis for the detection of "healthy" people who will develop AD for clinical trials. This will provide valuable information if it is feasible to use p-tau217 alone as inclusion criteria in this population.

Referee #3 (Remarks for Author):

Kira and colleagues investigated the predictive value of plasma p-tau217 for Alzheimer's disease compared to plasma p-tau181, NfL, and GFAP among 779 participants with up to 17 years of follow-up. They found that plasma p-tau217, NfL and GFAP had strong associations with AD risk within the first 9 years of follow-up, outperforming the performance of plasma p-tau181. In contrast, the predictive accuracy of p-tau217 was significantly reduced over the later years of follow-up. The Aβ misfolding biomarker measurement was performed in a subgroup analysis that demonstrated superior performance, especially as an early indicator of AD risk, many years before diagnosis. These findings offer new insights into the diagnostic performance of plasma biomarkers over the long course of AD. However, the reviewer has a few comments as follows:

1) The major concern is that the authors did not clearly describe how they defined AD and other types of dementia. Are those AD and control cases clinically defined or biologically defined? For Alzheimer's dementia patients, part of them may be amyloid negative, which may belong to non-AD cases, whereas around 20% of cognitively unimpaired older adults may be amyloid positive and at preclinical stages of AD. The authors should clearly describe these terms and make suitable statements for their results and conclusions.

2) The authors found similar diagnostic performance of p-tau217, NfL, and GFAP for AD, which may be due to the "AD" here represents Alzheimer's & Dementia rather than Alzheimer's disease. P-tau217 is a more AD-specific biomarker compared to GFAP and NfL.

3) "Inclusion of the Aβ misfolding biomarker to the p-tau217, p-tau181, GFAP, and NfL biomarker panel further improved predictive performance, particularly during later follow-up". It is very likely that Aβ misfolding occurs earlier in AD than tau and NfL. Can the authors explain why they reached better diagnostic performance as they included the Aβ misfolding biomarker in later follow-up?

4) Plasma p-Tau217 may be a superior biomarker in detecting Aβ plaque or tau tangle aggregation compared to plasma p-tau181, GFAP, and NfL, whereas plasma NfL may be a better biomarker to predict neurodegeneration and cognitive decline than other plasma biomarkers. Can the authors determine the diagnostic performance of plasma p-tau217, as well as other plasma biomarkers, in detecting amyloid positivity or tau positivity measured by either PET imaging or CSF biomarkers?

5) "Outliers were defined as > 4SDs from the mean for each of the blood-based 166 biomarkers and excluded from further analyses." Did the authors have any reason or reference for setting> 4 SDs as the outlier threshold?

Response to the Reviewers' comments:

Reviewer #1:

This manuscript evaluates the predictive value of plasma p-tau217 for Alzheimer's disease (AD) in a large, population-based prospective cohort with long follow-up, and compares its performance with other blood-based biomarkers (p-tau181, NfL, GFAP), as well as with an amyloid-β (Aβ) misfolding biomarker in a subgroup. The long follow-up (up to 17 years), prospective design, and availability of multiple biomarkers measured at baseline represent major strengths. The work addresses an important and timely question regarding the temporal utility of blood biomarkers across different stages of AD development.

General response
We thank the reviewer for the thorough review and for appreciating the strengths and timeliness of the study. We will answer all comments below.

Major comments

Point 1
A considerable proportion of samples initially selected for p-tau217 measurement were excluded due to technical failure or non-quantifiable concentrations (approximately 24% of samples with available material). The manuscript does not sufficiently address whether excluded participants differed systematically from those included (e.g., age, sex, dementia status, biomarker levels from prior assays). The authors should provide a comparison of baseline characteristics between included and excluded participants, and discuss how assay failure and missingness might bias hazard ratios and AUC estimates, particularly for long-term prediction.

Response
Samples were randomized across plates for the p-tau217 measurements to minimize potential bias related to plate assignment. Nevertheless, we agree that comparing baseline characteristics between included and excluded participants is informative. We have therefore added this comparison to Appendix Table S6, which shows that the two groups are comparable with respect to baseline characteristics, dementia incidences and biomarker levels from prior assays. Nevertheless, we acknowledge that any exclusion could theoretically influence effect estimates, particularly for long-term predicition and have added a statement addressing this point (page 19, lines 433-437).

Point 2
Although the study is based on a nested case-control sample, Cox proportional hazards models are applied without clear justification or weighting. It is unclear whether the sampling strategy preserves valid hazard ratio estimates for the underlying cohort, especially for subtype-specific dementia analyses. The authors should clarify whether and how the nested design was accounted for in the Cox model and justify the use of time-to-event analyses rather than conditional logistic regression, or provide sensitivity analyses demonstrating robustness of results.

Response
In our study, controls were randomly selected from the ESTHER cohort. Our primary objective was to assess the association between baseline biomarker levels and dementia incidence. Therefore, we used Cox proportional hazards models to incorporate the available time-to-event information. To assess robustness of results, we performed sensitivity analyses using logistic regression models for the association of p-tau217 with AD stratified by time of diagnosis. The results were consistent with those obtained by Cox models, supporting the robustness of our findings (Appendix Table S7, p.20 lines 459-461).

Point 3

The manuscript frequently describes p-tau217 as performing well "early," yet the strongest associations are observed within 0- 9 years prior to diagnosis, which may already correspond to prodromal or near-clinical disease rather than true preclinical AD. In contrast, the Aβ misfolding biomarker appears to perform better beyond 9 years. I suggest refining the conceptual framework distinguishing preclinical, prodromal, and near-clinical phases. Please, avoid overstating p-tau217's utility for "early" risk prediction when its performance declines substantially beyond 9 years.

Response

We have revised the terminology of early and late phases to prodromal and preclinical throughout the manuscript and tempered statements regarding p-tau217's utility for early risk prediction accordingly.

Point 4

Comparisons between p-tau217 and Aβ misfolding are based on a small subgroup (55 AD cases, 180 controls). Despite limited power, strong conclusions are drawn regarding the superiority of Aβ misfolding for long-term prediction. Please, emphasize the exploratory nature of these analyses and include wider discussion of uncertainty and potential instability of AUC estimates in small subgroups.

Response

We agree that the comparison between p-tau217 and Aß misfolding is based on a relatively small subgroup, which may limit statistical power and affect the stability of AUC estimates. We have therefore revised the manuscript in several places emphasizing the exploratory nature of these analyses and the associated uncertainty (p.7 lines 145 and 154; p.9 lines 188-189; p.12 lines 275-276; p.15 line 326; p.15 lines 339-341).

Point 5

Dementia diagnoses were GP-reported with heterogeneous diagnostic work-ups, and dementia subtypes were often unspecified. This limitation is acknowledged but not sufficiently integrated into the interpretation of subtype-specific findings (VD, MD). Please, discuss more explicitly how misclassification may differentially affect biomarker performance, particularly for p-tau217 specificity, and consider sensitivity analyses excluding mixed or unspecified cases, if feasible.

Response

We included a more detailed discussion in the interpretation of subtype-specific findings and performed a sensitivity analysis excluding mixed dementia cases from VD analyses (p.11 line 252 - p. 12 line 254, Appendix Table S5). This analysis yielded consistent results. A sensitivity analysis excluding unspecified cases is not feasible since analyses on VD and MD only included specified cases.

Point 6

While combined biomarker models yield high AUCs, it remains unclear how much incremental value p-tau217 adds beyond GFAP, NfL, and demographic/genetic variables. Please, provide formal measures of incremental predictive value (e.g., ΔAUC, net reclassification improvement, or decision-curve analysis) and clarify whether p-tau217 meaningfully improves clinical decision-making rather than statistical performance alone.

Response

We additionally included ΔAUC measures for AD in Appendix Table S2 (p.7, lines 151-152).

Ponit 7
Minor comments

1. Specify limits of detection and inter-/intra-assay variability for p-tau217.
2. Clarify handling of values below detection limits.
3. Report proportional hazards assumption testing.
4. Justify the choice of 9-year cut-point for follow-up stratification.
5. Some figures (ROC curves) are difficult to interpret due to overlapping lines; consider simplifying or moving additional curves to supplementary material
6. Ensure consistent terminology when referring to "prediction," "association," and "discrimination."
7. Moderate claims suggesting clinical readiness of p-tau217 for population screening, given observed limitations for long-term prediction.

Response

1. The limits of detection and inter-/intra-assay variability for p-tau217 were specified in the methods section (p.18, lines 399-402).
2. Handling of values below limits of detection was incluced in the Methods section (p.18, lines 402-403).
3. Proportional hazards assumption tests were added to the respective tables and figures.
4. The 9-year cut-point for follow-up stratification was chosen based on the approach used in the previous manuscript by Stocker et al. to ensure consistency and comparability with these previously published findings. We have clarified this rationale in the Methods section (p.20 line 449)
5. The figures have been enlarged and formatted according to the journal guidelines.
6. We revised the manuscript to ensure consistent terminology.
7. We have moderated the corresponding statements in the manuscript (p.13 lines 292-294).

Reviewer #2:

This study used a nested case-control design in the prospective ESTHER cohort with up to 17 years follow-up to test the predictive accuracy of different biomarkers, including p-tau217, for AD, vascular dementia and mixed dementia. They showed that p-tau217, NfL and GFAP have higher predictive accuracy within the first 9 years of follow-up while an amyloid aggregation assay has a higher predictive accuracy in later follow-ups. All in all, this is an intersting and important study that investigates the use of these biomarkers on a population-level, non-specialized dementia care setting. They use adequate statistical models with covariate corrections. However, I have the following converns, particularly regarding the interpretation of the usability of these biomarkers in cognitively unimpaired individuals. The following analyses should be added to further determine whether the general population should be target of these biomarkers:

General response
We thank the reviewer for the constructive review and highlighting the relevance of investigating blood-based biomarkers in a population-based, non-specialized dementia care setting. We will answer all comments below.

Point 1
- Dementia diagnosis and etiology are based on standardized questionnaires. However, it is not possible to verify this by the cognitive and biomarker status at the time of diagnosis. Also MCI is not included as

diagnosis which is the main target group for interventions. These should be included as important limitations.

Response

We included both arguments to the limitations (p.14 lines 315-317 and lines 321-323)

Point 2

- Are there any baseline characteristics regarding the cognitive status of the included individuals? It is important to emphasize if they were non-demented for the interpretation of the results.

Response

Given the relatively young age of participants at baseline (Mean (SD): 64.2 (6.5) years), it is unlikely that they had developed dementia by then. In addition, participants were asked to complete a self-administered questionnaire, which would not have been feasible for individuals with dementia. On top of that, the mental ability to participate was an inclusion crtieria of the ESTHER study and was assessed by the general practitioner at recruitment based on their knowledge of the patient. Therefore, we can assume that participants did not have dementia at baseline and that dementia became manifest and diagnosed during follow-up. Furthermore, dementia diagnosis dates were consistently documented by GPs and no case was before the baseline date. We have added the inclusion criteria of the ESTHER study to the manuscript (p.16 lines 350-352).

Point 3

- Please calculate the positive and negative predictive values for the different time episodes and biomarkers. I do not agree with the conclusion, especially lines 425-427. Likely, the PPV will show that p-tau217 is not suited as a screening tool in people without cognitive deficits. This interpretation would be in line with literature (e.g. PMID: 39533113, 37653254) showing that p-tau217 should be only used in people with MCI in the clinical setting.

Response

PPV and NPV are inherently dependent on both the selected biomarker cutoff and the underlying disease incidence. As both biomarker cutoffs and apparent incidence would be arbitrary in our study that used continuous biomarker measurements and a nested case-control design, we abstained from calculating PPVs and NPVs. However, we have revised the manuscript to reflect the more cautious interpretation rightfully requested by the reviewer, especially in the conclusion (p. 15, lines 339-341).

Point 4

- Please also include a power analysis for the detection of "healthy" people who will develop AD for clinical trials. This will provide valuable information if it is feasible to use p-tau217 alone as inclusion criteria in this population.

Response

Power strongly depends on sample size, the biomarker cutoff, the strength of the association and disease incidence. Although power calculations for our specific case-control setting would be of limited if any value for planned RCTs, the results of our study may inform potential power calculations for RCTs designed in populations of specific size and with specific incidence.

Reviewer #3:

Kira and colleagues investigated the predictive value of plasma p-tau217 for Alzheimer's disease compared to plasma p-tau181, NfL, and GFAP among 779 participants with up to 17 years of follow-up. They found that plasma p-tau217, NfL and GFAP had strong associations with AD risk within the first 9 years of follow-up, outperforming the performance of plasma p-tau181. In contrast, the predictive accuracy of p-tau217 was significantly reduced over the later years of follow-up. The Aβ misfolding biomarker measurement was performed in a subgroup analysis that demonstrated superior performance, especially as an early indicator of AD risk, many years before diagnosis. These findings offer new insights into the diagnostic performance of plasma biomarkers over the long course of AD. However, the reviewer has a few comments as follows:

General response
We thank the reviewer for his/her thoughtful review and for highlighting the importance of our findings for diagnostic performance of blood biomarkers for AD. We will answer all comments below.

Point 1
1) The major concern is that the authors did not clearly describe how they defined AD and other types of dementia. Are those AD and control cases clinically defined or biologically defined? For Alzheimer's dementia patients, part of them may be amyloid negative, which may belong to non-AD cases, whereas around 20% of cognitively unimpaired older adults may be amyloid positive and at preclinical stages of AD. The authors should clearly describe these terms and make suitable statements for their results and conclusions.

Response
Dementia diagnoses in this study were made heterogeneously based on reports from general practitioners, reflecting routine outpatient care in a population-based setting. As such, diagnoses were not collectively based on biomarker confirmation and amyloid status was not collected assessed in the cohort. While this is inherent to the design of the ESTHER study, we agree that it may result in some degree of misclassification, including the inclusion of amyloid-negative AD cases or preclinical amyloid-positive individuals among cognitively unimpaired participants. We adapted the respective section in the limitations respectively (p.14 lines 315-317) and also clarified this in the results and the methods section section (p.5, line 92 and p.16 lines 363-364).

Point 2
2) The authors found similar diagnostic performance of p-tau217, NfL, and GFAP for AD, which may be due to the "AD" here represents Alzheimer's & Dementia rather than Alzheimer's disease. P-tau217 is a more AD-specific biomarker compared to GFAP and NfL.

Response
We clearly distinguished between all-cause dementia and AD throughout the manuscript. Included AD cases were collectively indicated as such by their GPs. However, we have a high proportion of unspecified dementia cases in our study sample. This might have led to an underestimation of results for p-tau217 with regard to AD. This limitation has now been extensively discussed in the manuscript (p.14, lines 312-323).

Point 3
3) "Inclusion of the Aβ misfolding biomarker to the p-tau217, p-tau181, GFAP, and NfL biomarker panel further improved predictive performance, particularly during later follow-up". It is very likely that Aβ misfolding occurs earlier in AD than tau and NfL. Can the authors explain why they reached better diagnostic performance as they included the Aβ misfolding biomarker in later follow-up?

Response

We would like to clarify that in our study, biomarker measurements were obtained from baseline blood-samples, and follow-up periods refer to the time from baseline to diagnosis. Thus, cases occurring in the late follow-up period represent individuals with a longer interval between biomarker assessment and diagnosis. In this context, Aß misfolding may capture early pathological changes that are not yet fully reflected by p-tau217, p-tau181, GFAP and NfL, thereby providing complementary information and improving predictive performance in this time window. However, it has to be noted that Aß analyses were restricted to a subgroup with a limited number of AD cases (controls: n=180; $AD_{0\text{-}9\ years}$ n=31; $AD_{9\text{-}17\ years}$ n=24; $AD_{0\text{-}17\ years}$ n=55). In particular, the small number of events in the later time window limits the robustness of these estimates. This has been clarified in the course of the revision and the respective findings were interpreted with greater caution.

Point 4

4) Plasma p-Tau217 may be a superior biomarker in detecting Aβ plaque or tau tangle aggregation compared to plasma p-tau181, GFAP, and NfL, whereas plasma NfL may be a better biomarker to predict neurodegeneration and cognitive decline than other plasma biomarkers. Can the authors determine the diagnostic performance of plasma p-tau217, as well as other plasma biomarkers, in detecting amyloid positivity or tau positivity measured by either PET imaging or CSF biomarkers?

Response

We thank the reviewer for this important suggestion. However, due to the population-based nature of this cohort study, the collection of CSF samples or performance of PET imaging was not feasible and out of the scope of the ESTHER study. Therfore, analyses of the diagnostic performance of plasma biomarkers for detecting amyloid or tau positivity cannot be performed in the present dataset.

Point 5

5) "Outliers were defined as > 4SDs from the mean for each of the blood-based 166 biomarkers and excluded from further analyses." Did the authors have any reason or reference for setting> 4 SDs as the outlier threshold?

Response

Outliers were defined as >4SDs in accordance with the approach used in the previous manuscript by Stocker et al. This threshold was applied to ensure consistency with previously published analyses and comparability of results. We have clarified this in the methods section of the manuscript (page 20, line 449).

3rd Jun 2026

Dear Prof. Brenner,

Thank you for the submission of your revised manuscript to EMBO Molecular Medicine. We have now received the enclosed reports from the two reviewers who agreed to reassess your manuscript. As you will see, both referees agree that the revision has improved the study, although both still raise important concerns regarding overstatement and request more accurate, cautious wording for several key conclusions.

Before we can accept your manuscript, pending the amendments outlined below, please address the remaining comments from the two reviewers. Please use track changes, as we need to review the modifications. Please also provide a point-by-point response to the remaining reviewer comments.

On a more editorial level:

1. Please note that Figure 1 should not be called out after Figure 7. Please add callouts for the individual panels of Figures 3-7, and add a missing callout for Appendix Figure S5.

2. References: "et al" needs to be used after 10 author names.

3. The Appendix should be provided as a PDF. Highlighting and track changes in the Appendix should be removed, as this file will not be proofread.

4."The Paper Explained" should be moved to the manuscript file, and the three section headings "Problem", "Results", and "Impact" should be incorporated.

5. The synopsis image should be a visual abstract style, rather than an actual figure from the paper. Please provide a new version as a separate PNG file with exact dimensions of 550 px (width) × 300 px (height).

6. Remove "data not shown" (page 10). As per our guidelines, on "Unpublished Data" the journal does not permit citation of "Data not shown". All data referred to in the paper should be displayed in the main or Expanded View figures.

7. Data availability- remove the currently provided statement and use the following: "This study includes no data deposited in external repositories."

8. Please enter the funding information in the online submission system as well as in the Acknowledgements section. Please ensure that the information is consistent.

9. Please address the following issues in figure legends:
- Please note that the exact p values are not provided in the legends of figures S1A, B
- Please note that information related to n is missing in the legends of figures S1 A-D;

10. Please correct the order and headings of the sections in the manuscript text file to: Abstract / The Paper Explained /Introduction / Results / Discussion / Methods / Data Availability / Acknowledgements / Disclosure and Competing Interests Statement / References / Figure Legends / Tables / Expanded View Figure Legends

I look forward to reading a new revised version of your manuscript as soon as possible.

Kind regards,
Jingyi

Jingyi Hou
Senior Editor
EMBO Molecular Medicine

*** Instructions to submit your revised manuscript ***

*** PLEASE NOTE *** As part of the EMBO Publications transparent editorial process initiative (see our Editorial at https://www.embopress.org/doi/pdf/10.1002/emmm.201000094), EMBO Molecular Medicine will publish online a Review Process File to accompany accepted manuscripts.

In the event of acceptance, this file will be published in conjunction with your paper and will include the anonymous referee reports, your point-by-point response and all pertinent correspondence relating to the manuscript. If you do NOT want this file to be published, please inform the editorial office at contact@embomolmed.org.

***** Reviewer's comments *****

Referee #2 (Remarks for Author):

I thank the authors for the revised manuscript. I mostly agree with their argumentation and appreciate their expanded limitations sections. I have one remaining concern which should be at least discussed in more detail. Due to the nested case-control study design, as the authors rightfully say in their response letter, it is not possible to calculate true positive or negative predictive values. The case-control study likely overestimates the AD prevalence. However they still have a very low AD incidence, which will result in a very low negative predictive value. Therefore I do not agree with their conclusion that p-tau217 can be used on a population-wide scale (l. 338). Along these lines they should tone down their conclusions in the "Prevention" and "Clinical relevance of the findings" sections. It is important to emphasize that p-tau217 from the blood is a biomarker to screen for the etiology in people who are cognitively impaired or at least have subjective cognitive impairment.

Referee #3 (Remarks for Author):

Well, the reviewer appreciates the authors' revisions and responses to the comments on the initial version of the manuscript. However, the reviewer does not think it is suitable to claim "Predictive value of plasma p-tau217 for Alzheimer's disease compared to other blood-based biomarkers" if the authors cannot provide evidence for those individuals' biological status. They can only say Alzheimer's disease and related dementia or Alzheimer's dementia. The authors should revise all the descriptions for Alzheimer's disease throughout the manuscript, including the title, abstract, and main text.

Response to the Reviewers' comments:

Referee #2 (Remarks for Author):

I thank the authors for the revised manuscript. I mostly agree with their argumentation and appreciate their expanded limitations sections. I have one remaining concern which should be at least discussed in more detail. Due to the nested case-control study design, as the authors rightfully say in their response letter, it is not possible to calculate true positive or negative predictive values. The case-control study likely overestimates the AD prevalence. However they still have a very low AD incidence, which will result in a very low negative predictive value. Therefore I do not agree with their conclusion that p-tau217 can be used on a population-wide scale (l. 338). Along these lines they should tone down their conclusions in the "Prevention" and "Clinical relevance of the findings" sections. It is important to emphasize that p-tau217 from the blood is a biomarker to screen for the etiology in people who are cognitively impaired or at least have subjective cognitive impairment.

Response

We thank the reviewer for this valuable comment. We have revised and toned down the conclusions in both the “Clinical relevance of the findings” (p.14, lines 308-316) and the “Prevention” (p.15, lines 325-330) sections and now emphasize the potential role of blood-based biomarkers for risk stratification and etiological assessment rather than population wide screening (p.16, line 361).

Referee #3 (Remarks for Author):

Well, the reviewer appreciates the authors' revisions and responses to the comments on the initial version of the manuscript. However, the reviewer does not think it is suitable to claim "Predictive value of plasma p-tau217 for Alzheimer's disease compared to other blood-based biomarkers" if the authors cannot provide evidence for those individuals' biological status. They can only say Alzheimer's disease and related dementia or Alzheimer's dementia. The authors should revise all the descriptions for Alzheimer's disease throughout the manuscript, including the title, abstract, and main text.

Response

We thank the reviewer for this important comment. We agree that our outcome is based on clinical diagnoses rather than biomarker-confirmed Alzheimer’s disease. We have therefore revised the manuscript throughout and now consistently use the term “Alzheimer’s dementia”.

26th Jun 2026

Dear Prof. Brenner,

Thank you for sending us your revised article. We are pleased to inform you that your manuscript is accepted for publication and is now being sent to our publisher to be included in the next available issue of EMBO Molecular Medicine.

Your manuscript will be processed for publication by EMBO Press. It will be copy edited and you will receive page proofs prior to publication. Please note that you will be contacted by Springer Nature Author Services to complete licensing and payment information.

You may qualify for financial assistance for your publication charges - either via a Springer Nature fully open access agreement or an EMBO initiative. Check your eligibility: https://link.springer.com/journal/44321/how-to-publish-with-us

Should you be planning a Press Release on your article, please notify the production team via this form in order to coordinate publication and release dates: https://forms.cloud.microsoft/e/eH4A8JVP6g

If you have any questions, please do not hesitate to contact the Editorial Office. Thank you for your contribution to EMBO Molecular Medicine.

Kind regards,
Jingyi

Jingyi Hou
Senior Editor
EMBO Molecular Medicine

>>> Please note that it is EMBO Molecular Medicine policy for the transcript of the editorial process (containing referee reports and your response letter) to be published as an online supplement to each paper. If you do NOT want this, you will need to inform the Editorial Office via email immediately. More information is available here: https://link.springer.com/partners/embo-press/editorial-policies#Peer%20review

EMBO Press Author Checklist

Corresponding Author Name: Prof. Dr. Hermann Brenner	
Journal Submitted to: EMBO Molecular Medicine	
Manuscript Number: EMM-2026-23356	

USEFUL LINKS FOR COMPLETING THIS FORM
The EMBO Journal - Author Guidelines
EMBO Reports - Author Guidelines
Molecular Systems Biology - Author Guidelines
EMBO Molecular Medicine - Author Guidelines

Reporting Checklist for Life Science Articles (updated January
This checklist is adapted from Materials Design Analysis Reporting (MDAR) Checklist for Authors. MDAR establishes a minimum set of requirements in transparent reporting in the life sciences (see Statement of Task: 10.31222/osf.io/9sm4x). Please follow the journal's guidelines in preparing your
Please note that a copy of this checklist will be published alongside your article.

Abridged guidelines for figures
1. Data
The data shown in figures should satisfy the following conditions:
- the data were obtained and processed according to the field's best practice and are presented to reflect the results of the experiments in an accurate and unbiased manner.
- ideally, figure panels should include only measurements that are directly comparable to each other and obtained with the same assay.
- plots include clearly labeled error bars for independent experiments and sample sizes. Unless justified, error bars should not be shown for technical
- if n<5, the individual data points from each experiment should be plotted. Any statistical test employed should be justified.
- Source Data should be included to report the data underlying figures according to the guidelines set out in the authorship guidelines on Data

2. Captions
Each figure caption should contain the following information, for each panel where they are relevant:
- a specification of the experimental system investigated (eg cell line, species name).
- the assay(s) and method(s) used to carry out the reported observations and measurements.
- an explicit mention of the biological and chemical entity(ies) that are being measured.
- an explicit mention of the biological and chemical entity(ies) that are altered/varied/perturbed in a controlled manner.
- the exact sample size (n) for each experimental group/condition, given as a number, not a range;
- a description of the sample collection allowing the reader to understand whether the samples represent technical or biological replicates (including how many animals, litters, cultures, etc.).
- a statement of how many times the experiment shown was independently replicated in the laboratory.
- definitions of statistical methods and measures:
  - common tests, such as t-test (please specify whether paired vs. unpaired), simple χ2 tests, Wilcoxon and Mann-Whitney tests, can be unambiguously identified by name only, but more complex techniques should be described in the methods section;
  - are tests one-sided or two-sided?
  - are there adjustments for multiple comparisons?
  - exact statistical test results, e.g., P values = x but not P values < x;
  - definition of 'center values' as median or average;
  - definition of error bars as s.d. or s.e.m.

Please complete ALL of the questions below.
Select "Not Applicable" only when the requested information is not relevant for your study.

Materials

Newly Created Materials	Information included in the manuscript?	In which section is the information available? (Reagents and Tools Table, Materials and Methods, Figures, Data Availability Section)
New materials and reagents need to be available; do any restrictions apply?	Not Applicable	

Antibodies	Information included in the manuscript?	In which section is the information available? (Reagents and Tools Table, Materials and Methods, Figures, Data Availability Section)
For **antibodies** provide the following information:<br>- Commercial antibodies: RRID (if possible) or supplier name, catalogue number and or/clone number<br>- Non-commercial: RRID or citation	Not Applicable	

DNA and RNA sequences	Information included in the manuscript?	In which section is the information available? (Reagents and Tools Table, Materials and Methods, Figures, Data Availability Section)
**Short novel DNA or RNA including primers, probes**: provide the sequences.	Not Applicable	

Cell materials	Information included in the manuscript?	In which section is the information available? (Reagents and Tools Table, Materials and Methods, Figures, Data Availability Section)
**Cell lines:** Provide species information, strain. Provide accession number in repository **OR** supplier name, catalog number, clone number, and/**OR** RRID.	Not Applicable	
**Primary cultures:** Provide species, strain, sex of origin, genetic modification status.	Not Applicable	
Report if the cell lines were recently **authenticated** (e.g., by STR profiling) and tested for mycoplasma contamination.	Not Applicable	

Experimental animals	Information included in the manuscript?	In which section is the information available? (Reagents and Tools Table, Materials and Methods, Figures, Data Availability Section)
**Laboratory animals or Model organisms:** Provide species, strain, sex, age, genetic modification status. Provide accession number in repository **OR** supplier name, catalog number, clone number, **OR** RRID.	Not Applicable	
**Animal observed in or captured from the field:** Provide species, sex, and age where possible.	Not Applicable	
Please detail **housing and husbandry conditions**.	Not Applicable	

Plants and microbes	Information included in the manuscript?	In which section is the information available? (Reagents and Tools Table, Materials and Methods, Figures, Data Availability Section)
**Plants:** provide species and strain, ecotype and cultivar where relevant, unique accession number if available, and source (including location for collected wild specimens).	Not Applicable	
**Microbes:** provide species and strain, unique accession number if available, and source.	Not Applicable	

Human research participants	Information included in the manuscript?	In which section is the information available? (Reagents and Tools Table, Materials and Methods, Figures, Data Availability Section)
If collected and within the bounds of privacy constraints report on age, sex and gender or ethnicity for all study participants.	Yes	Materials and Methods, Results, Table 1

Core facilities	Information included in the manuscript?	In which section is the information available? (Reagents and Tools Table, Materials and Methods, Figures, Data Availability Section)
If your work benefited from core facilities, was their service mentioned in the acknowledgments section?	Not Applicable	

Design

Study protocol	Information included in the manuscript?	In which section is the information available? (Reagents and Tools Table, Materials and Methods, Figures, Data Availability Section)
If study protocol has been **pre-registered, provide DOI in the manuscript**. For clinical trials, provide the trial registration number **OR** cite DOI.	Not Applicable	
Report the **clinical trial registration number** (at ClinicalTrials.gov or equivalent), where applicable.	Not Applicable	

Laboratory protocol	Information included in the manuscript?	In which section is the information available? (Reagents and Tools Table, Materials and Methods, Figures, Data Availability Section)
Provide DOI OR other citation details if **external detailed step-by-step protocols** are available.	Not Applicable	

Experimental study design and statistics	Information included in the manuscript?	In which section is the information available? (Reagents and Tools Table, Materials and Methods, Figures, Data Availability Section)
Include a statement about **sample size** estimate even if no statistical methods were used.	Not Applicable	
Were any steps taken to minimize the effects of subjective bias when allocating animals/samples to treatment (e.g. **randomization procedure**)? If yes, have they been described?	Yes	Materials and Methods
Include a statement about **blinding** even if no blinding was done.	Not Applicable	
Describe **inclusion/exclusion criteria** if samples or animals were excluded from the analysis. Were the criteria pre-established? If sample or data points were omitted from analysis, report if this was due to attrition or intentional exclusion and provide justification.	Yes	Materials and Methods
For every figure, are **statistical tests** justified as appropriate? Do the data meet the assumptions of the tests (e.g., normal distribution)? Describe any methods used to assess it. Is there an estimate of variation within each group of data? Is the variance similar between the groups that are being statistically compared?	Yes	Materials and Methods

Sample definition and in-laboratory replication	Information included in the manuscript?	In which section is the information available? (Reagents and Tools Table, Materials and Methods, Figures, Data Availability Section)
In the figure legends: state number of times the experiment was **replicated** in laboratory.	Not Applicable	
In the figure legends: define whether data describe **technical or biological replicates**.	Not Applicable	

Ethics

Ethics	Information included in the manuscript?	In which section is the information available? (Reagents and Tools Table, Materials and Methods, Figures, Data Availability Section)
Studies involving **human participants**: State details of **authority granting ethics approval** (IRB or equivalent committee(s), provide reference number for approval.	Yes	Materials and Methods
Studies involving **human participants**: Include a statement confirming that **informed consent** was obtained from all subjects and that the experiments conformed to the principles set out in the WMA Declaration of Helsinki and the Department of Health and Human Services Belmont Report.	Yes	Materials and Methods
Studies involving **human participants: For** publication of **patient photos**, include a statement confirming that consent to publish was obtained.	Not Applicable	
Studies involving experimental **animals**: State details of **authority granting ethics approval** (IRB or equivalent committee(s), provide reference number for approval. Include a statement of compliance with ethical regulations.	Not Applicable	
Studies involving **specimen and field samples:** State if relevant **permits** obtained, provide details of authority approving study; if none were required, explain why.	Not Applicable	

Dual Use Research of Concern (DURC)	Information included in the manuscript?	In which section is the information available? (Reagents and Tools Table, Materials and Methods, Figures, Data Availability Section)
Could your study fall under dual use research restrictions? Please check biosecurity documents and list of **select agents and toxins** (CDC): https://www.selectagents.gov/sat/list.htm	Not Applicable	
If you used a select agent, is the security level of the lab appropriate and reported in the manuscript?	Not Applicable	
If a study is subject to dual use research of concern regulations, is the name of the **authority granting approval and reference number** for the regulatory approval provided in the manuscript?	Not Applicable	

Reporting

The MDAR framework recommends adoption of discipline-specific guidelines, established and endorsed through community initiatives. Journals have their own policy about requiring specific guidelines and recommendations to complement MDAR.

Adherence to community standards	Information included in the manuscript?	In which section is the information available? (Reagents and Tools Table, Materials and Methods, Figures, Data Availability Section)
State if relevant guidelines or checklists (e.g., **ICMJE, MIBBI, ARRIVE, PRISMA**) have been followed or provided.	Not Applicable	
For **tumor marker prognostic studies,** we recommend that you follow the **REMARK** reporting guidelines (see link list at top right). See author guidelines, under 'Reporting Guidelines'. Please confirm you have followed these guidelines.	Not Applicable	
For **phase II and III randomized controlled trials**, please refer to the **CONSORT** flow diagram (see link list at top right) and submit the CONSORT checklist (see link list at top right) with your submission. See author guidelines, under 'Reporting Guidelines'. Please confirm you have submitted this list.	Not Applicable	

Data Availability

Data availability	Information included in the manuscript?	In which section is the information available? (Reagents and Tools Table, Materials and Methods, Figures, Data Availability Section)
Have **primary datasets** been deposited according to the journal's guidelines (see 'Data Deposition' section) and the respective accession numbers provided in the Data Availability Section?	Yes	Data Availability Section
Were **human clinical and genomic datasets** deposited in a public access-controlled repository in accordance to ethical obligations to the patients and to the applicable consent agreement?	Not Applicable	
Are **computational models** that are central and integral to a study available without restrictions in a machine-readable form? Were the relevant accession numbers or links provided?	Not Applicable	
If publicly available data were reused, provide the respective **data citations in the reference list**.	Not Applicable	
